# Minimum Cost Deployment of Bistatic Radar Sensor for Perimeter Barrier Coverage

**DOI:** 10.3390/s19020225

**Published:** 2019-01-09

**Authors:** Xianghua Xu, Chengwei Zhao, Tingcong Ye, Tao Gu

**Affiliations:** 1 School of Computer Science and Technology, Hangzhou Dianzi University, Hangzhou 310018, China; zcw@hdu.edu.cn (C.Z.); tingcong.ye@hdu.edu.cn (T.Y.); 2 Department of Computer Science, RMIT University, Melbourne, VIC 3001, Australia; tao.gu@rmit.edu.au

**Keywords:** circle barrier coverage, bistatic radar sensor, minimum cost placement

## Abstract

Perimeter barriers can provide intrusion detection for a closed area. It is efficient for practical applications, such as coastal shoreline monitoring and international boundary surveillance. Perimeter barrier coverage construction in some regions of interest with irregular boundaries can be represented by its minimum circumcircle and every point on the perimeter can be covered. This paper studies circle barrier coverage in Bistatic Radar Sensor Network (BRSN) which encircles a region of interest. To improve the coverage quality, it is required to construct a circle barrier with a predefined width. Firstly, we consider a BR deployment problem to constructing a single BR circular barrier with minimum threshold of detectability. We study the optimized BR placement patterns on the single circular ring. Then the unit costs of the BR sensor are taken into account to derive the minimum cost placement sequence. Secondly, we further consider a circular BR barrier with a predefined width, which is wider than the breadth of Cassini oval sensing area with minimum threshold of detectability. We propose two segment strategies to efficiently divide a circular barrier to several adjacent sub-ring with some appropriate width: Circular equipartition strategy and an adaptive segmentation strategy. Finally, we propose approximate optimization placement algorithms for minimum cost placement of BR sensor for circular barrier coverage with required width and detection threshold. We validate the effectiveness of the proposed algorithms through theory analysis and extensive simulation experiments.

## 1. Introduction

Barrier Coverage is an important sensor deployment issue in many industrial, consumer, and military applications, such as machine management, health care monitoring, battlefield surveillance [1], etc. Recent years have witnessed a trend that radar sensors have been increasingly deployed in guarding militarized zones, and monitoring hazards warehouse and frontier [2,3,4,5]. Traditional passive sensors typically leverage on a disk sensing model. In contrast, Bistatic Radar (BR) sensors use a Cassini oval sensing model [6]. The sensing region of a BR sensor depends on the locations of both the BR transmitter and receiver, and is characterized by a Cassini oval. Moreover, since a BR transmitter (or receiver) can potentially form multiple BRs with different BR transmitters (or receivers, respectively), the sensing regions of different BRs are coupled, making the coverage of a BR network (BRN) highly non-trivial.

Some recent works on BR sensor coverage are mainly focused on line barrier. In [7], a minimum cost placement algorithm was developed for line barrier coverage. To improve quality, a belt with a predefined breadth is considered and covered by several same lines. In [8], the authors studied both fault tolerance and energy-saving issues combining the minimum cost placement of BR sensors for belt barrier coverage. They focus only on the line-based barrier coverage that cannot be completely applied to other application scenarios, such as perimeter barrier which can encircle the whole protected region.

Perimeter barrier consisting of BR sensors can provide intrusion detection for a closed area. It is efficient for practical applications, such as coastal shorelines monitoring and international boundary surveillance. Perimeter barrier coverage construction in some regions of interest whose irregular boundary can be represented by its minimum circumcircle and bistatic radars are to be deployed on the circumcircle perimeter to construct a perimeter barrier coverage such that every point on the perimeter can be covered [6]. Given the characteristics of a Cassini oval model, it is challenging to optimize the radar sensors deployment in the circle perimeter barrier coverage. In addition, to improve coverage quality, we need to construct several circular BR barriers which form a circular ring with some width to achieve higher reliability of monitoring. Moreover, to efficiently construct a cost-efficient circle barrier, the unit costs of the BR transmitter and receiver also should be considered in the barrier optimization stage. These factors in the real applications affect the design of barrier coverage algorithms or models.

In this paper, we study a minimum cost BR sensor placement algorithm to construct a circular BR coverage with a predefined breadth, to improve coverage quality of perimeter monitoring for a protected area with minimum threshold of detectability. Firstly, we consider a BR deployment problem to constructing a single BR circular barrier with minimum threshold of detectability. We study and prove the optimized BR placement patterns on the single circular ring. Then the unit costs of the BR sensor are taken into account to derive the minimum cost placement sequence. Secondly, to enhance the coverage quality, we consider an annulus BR sensor barrier with a predefined breadth, which is wider than the breadth of Cassini oval sensing area with minimum threshold of detectability. In [7], the authors study the BR deployment scheme of line barrier and how to construct a belt coverage with predefined width. But how to efficiently segment an annulus ring to several adjacent sub-rings with some appropriate width so as to ensure the minimum cost annulus BR barrier coverage is also challenging. We propose two segment strategies: (1) A circular equipartition strategy such that the BR sensors deployed on a circle can form a barrier with some breadth, and one or more such circles can form an annulus barrier with the required breadth; and (2) an adaptive segmentation strategy such that multiple circle barriers with appropriate breadths can optimum form an annulus barrier with the required breadth. Finally, we propose optimization algorithm to optimize the segmentation of circular ring and BR placement pattern on each circle to minimize the total placement cost, and to achieve approximate optimal circular coverage with predefined width and detectability.

The proposed algorithms are validated by simulation results. To the best of our knowledge, this appears the first paper to investigate the minimum cost circle barrier coverage problem. The main contributions of our work are summarized as follows.

- Firstly, we investigate the Cassini oval sensing models and discuss a variety of barrier coverage cases. We discover and prove the optimized BR placement patterns and sequence on a circular ring. Then the unit costs of the BR sensor are taken into accounts to derive the minimum cost placement sequence, which is then used to design an algorithm for any minimum cost placement in circle-based barrier construction.
- Secondly, we further study the optimal BR placement on an circular barrier with a predefined breadth. We propose two division strategies, circular equipartition strategy and an adaptive segmentation strategy, to segment an annulus ring to several adjacent sub-ring with appropriate width so as to ensure the minimum cost annulus BR barrier coverage with required detection threshold.
- Finally, we propose approximate optimization placement algorithms for minimum cost placement of BR sensor for annulus barrier coverage with required width and detection threshold. We validate the effectiveness of the proposed algorithms through extensive simulation experiments.

The rest of paper is organized as follows: Section 2 reviews the related work. Section 3 introduces the system model and problem description. Section 4 describes the optimal placement sequence on the single circle-based barrier and Section 5 provides the solution to the minimum placement cost problem and simulation experimente. Section 6 concludes the paper.

## 2. Related Work

Barrier coverage is an important issue in many wireless sensor network applications, such as border intrusion detection and environmental safety monitoring. We review the related work in barrier coverage based on the following three models—the disk cover model [9,10,11,12,13,14], the sector cover model [15,16,17,18], and the BR sensor cover model [6,19,20].

We first review the work leveraging on the disk cover model. In [9], the authors considered the barrier coverage problem of heterogeneous wireless sensor networks, and used Helly’s Theorem to solve the problem of k-coverage based on heterogeneous sensors and data acquisition. The authors in [10] described a new deployment method of probabilistic sensor to improve network coverage. In [11], the maximum life scheduling of target coverage and data collection was proposed. In [12], to address the problem of maximizing the life cycle of barrier coverage, the authors proposed two sleep-wake scheduling algorithms named Stint and Prahari, and then proposed three enhanced algorithms, i.e., Greedy-Cover-Eraser, Greedy-Edge-Eraser, and MaxFlow-Edge-Eraser. In [13], the authors firstly introduced the concept of strong and weak barrier coverage, and then deduced the critical conditions to construct weak barrier coverage. The authors in [14] introduced the concept of local barrier coverage nad proposed a local algorithm a protocol to maximize the life of barrier coverage. In [21], the authors introduced a heterogeneous barrier-coverage in which guarantees that any penetration variation of intruder is detected by at least one sensor with different sensing capabilities. The authors in [22] considered the problem of intrusion in transversal directions. They introduced the concept of crossed barrier coverage which can prevent an intruder from crossing the entire target area from a specific direction without being detected. In addition, they also proposed a multi-round shortest path algorithm (MSPA) to solve the optimization problem, which works heuristically to guarantee efficiency while maintaining near-optimal solutions. The authors in [23] introduced a new type of barrier, virtual emotion barrier, which is able to detect emotion by devices with wireless signal in Internet of Things (IoT) environment. They formally defined a problem whose objective is to construct virtual emotion barrier in the given area including IoT devices such that the detection accuracy of emotion by virtual emotion barrier is maximized. To solve the problem, they proposed a greedy-emotion-accuracy approach.

We now move to the studies leveraging on the sector cover model. The authors in [15] proposed a polynomial-time algorithm to determine the orientation of the sensors to build strong barrier coverage. In [16], the authors mainly adopted the sector coverage model and guaranteed the communication of the whole network by constructing connected dominating set. In [17], the authors proposed a distributed β-breadth belt-barrier construction algorithm without rotation (D-TriB) to improve the image quality in the wireless vision sensor network. In [18], the authors studied the exposure path prevention problem based on directional sensor network coverage, and mapped the exposure path problem into the sector-based percolation model, and then deployed the critical density boundary of the directional sensors according to the two-dimensional Poisson process.

Some recent work considers the problem of mobile sensor coverage. For example, in [24], the authors deployed a sensor network which consists of both mobile and static sensors, and the mobile sensors can move from a dense area to a sparse area to improve coverage rate. In [25], the authors proposed a distributed algorithm for building k-coverage based on mobile sensors. In [26], a fully distributed algorithm based on virtual force and convex analysis is developed for the objective to relocate the sensors from the original positions to uniformly distribute on the convex hull of the region to building the barrier. In [6], the authors discussed how to construct a barrier on the smallest circumcircle of the area which is protected to minimize the cost. At the same time, the minimum matching algorithm is given to calculate the minimum total moving distance of the barrier formed by mobile sensors. In [27], the authors introduced a new architecture of barrier, event-driven partial barrier, which is able to monitor any movements of objects in the event-driven environment. Also, a resilient event-driven partial barrier is introduced to consider the case that the constructed barriers collapsed due to failures of some sensors consisting of those barriers. We know that existing works on barrier coverage typically assume that sensor nodes have accurate location information, which is not reasonable or practical for many real sensor networks. In [28], the authors studied the barrier coverage problem when sensor nodes have location errors and deploy mobile sensor nodes to improve barrier coverage if the network is not barrier-covered after initial deployment. They analyzed the effects of location errors for barrier coverage and propose a fault-tolerant weighted barrier graph to model the barrier coverage formation problem.

Recently, the technology of BR sensor began to receive wider attention. In [19], the authors considered the problem of deploying a network of BRs in a region to maximize the worst-case intrusion detectability, while minimizing the vulnerability of a barrier. In [20], the authors proposed a random Voronoi algorithm to calculate the optimal position of transmitters and receivers so that the maximum distance between all the points of interest in the area to their nearest sensor pair is minimized.

The general problem of barrier coverage does not require the coverage width of the barrier, but only needs to ensure that the behavior of the object across the boundary can be monitored. For example, in [6], to protect an irregular region, the author selected the boundary of the smallest circumscribed circle of the region to construct a barrier, and proposed a matching algorithm to calculate the minimum cost of resetting sensors under the condition of disrupting the sensor sequence. Since there is only one barrier, it has a great potential safety hazard for intruder invaders and emergencies. On the basis of this, in [7], the authors discussed the problem of straight-line barrier with a certain width and proposed a corresponding effective solution. Our work is motivated by the first two papers, but differs in several aspects. First, we focus on different background of the problem. We address the problem of annulus barrier coverage which in particular poses an unprecedented challenge compared to the traditional background of barrier coverage. Secondly, the calculation of the effective width and length will be more challenging than that in the straight barrier coverage, requiring an innovative solution to the boundary problem of the circle. In addition, we consider minimum cost coverage which is essential to real deployment. Our intuition is to divide the annulus barrier into a number of circles. Since each circle’s radius is different, it is difficult to achieve the complete coverage while minimizing cost.

## 3. Sensor Model and Problem Description

We define some variables used in the paper in Table 1.

### 3.1. Sensor Model

In the BR sensor model, the transmitter and sensor can be placed in different positions. Let $\bar{XY}$ denote the line segment between point *X* and point *Y*. ∥XY∥ denotes the European distance of the line segment, and $T_{i}$ and $R_{j}$ denote transmitter $T_{i}$ and receiver $R_{j}$, respectively.

According to [29], for a pair of BR sensor $T_{i}-R_{j}$, the signal-to-noise ratio (SNR) of a monitocircular point can be computed as follows:

(1) $$SNR(A)=\frac{K}{{∥T_{i}A∥}^{2}\cdot {∥R_{j}A∥}^{2}}$$

Here, *K* is a constant determined by the physical properties of the BR sensor, such as the energy of sensor, the radar sensor cross section and the antenna’s power gain. $∥T_{i}A∥$ represents the European distance of the monitocircular point *A* to transmitter $T_{i}$. $∥R_{j}A∥$ represents the European distance from the monitocircular point *A* to receiver $R_{j}$.

In a Bistatic Radar Sensor Network (BRSN), we assume that all sensors operate at an orthogonal frequency [30,31] to avoid mutual interference between receivers. We also assume that all the transmitters and receivers have the same physical attributes. Because a transmitter can match multiple receivers, a receiver can also match multiple transmitters. In the fourth part, we only consider receiving the signal from the nearest two transmitters for a receiver. Consider circular the signal-to-noise ratio (SNR) of any monitocircular points *A*, according to [19,32], we can select the maximum SNR for this point as its SNR, as it can be monitored by many pairs of radar sensors. We define $SNR_{A}^{\max}$ as follows.

(2) $$SNR_{A}^{\max}=\max_{\left(T_{i},R_{j}\right)}\frac{K}{{∥T_{i}A∥}^{2}\cdot {∥R_{j}A∥}^{2}}$$

Thus, if a point of interest *A* can be monitored, then $SNR_{A}^{\max}\geq \varepsilon$ should be satisfied, where ε is the given threshold of SNR. We set the SNR threshold of the barrier to ε.

For convenience in the paper, we introduce the concept of detectability where the detectability of a point *A* is described as follows.

(3) $$l(A)=\min_{\left(T_{i},R_{j}\right)}∥T_{i}A∥\cdot ∥R_{j}A∥$$

Consider the requirement of minimum threshold, we obatin the threshold of detectability as:

(4) $$l_{\max}^{2}=\sqrt{\frac{K}{\varepsilon }}$$

where ε is the given threshold ε of SNR. It is clear that we need to satisfy $l\left(A\right)\leq l_{\max}^{2}$ to make the point of interest *A* be covered, which is equivalent to $SNR_{A}^{\max}\geq \varepsilon$.

**Figure 1:**
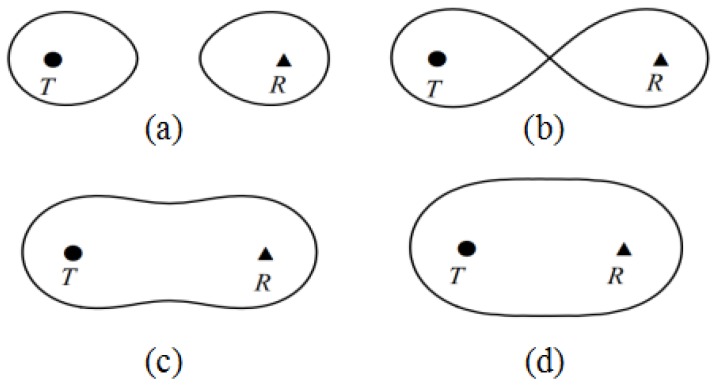
(**a**) $d\left(T,R\right)>2l_{\max}$; (**b**) $d\left(T,R\right)=2l_{\max}$; (**c**) $\sqrt{2}l_{\max}<d(T,R)<2l_{\max}$; (**d**) $0<d(T,R)<\sqrt{2}l_{\max}$.

Under the given threshold of SNR, according to [9], with change in the distance between transmitter and receiver sensor, the coverage area of the BR sensor model can be divided into four cases, as shown in Figure 1.

(*a*)  $d\left(T,R\right)>2l_{\max}$, the coverage area is two disjointed parts;
(*b*)  $d\left(T,R\right)=2l_{\max}$, the coverage area is surrounded by the Bernoulli double new line;
(*c*)  $\sqrt{2}l_{\max}<d\left(T,R\right)<2l_{\max}$, the coverage area is surrounded by the waist closed curve;
(*d*)  $0<d\left(T,R\right)<\sqrt{2}l_{\max}$, the coverage area is surrounded by the elliptical curve,
  where d(T,R) represents the distance between the transmitter and the receiver.

For the particular problem we study in this paper, we need to consider three sensor coverage cases, i.e., (*a*), (*c*), and (*d*).

### 3.2. Problem Definition

**Figure 2:**
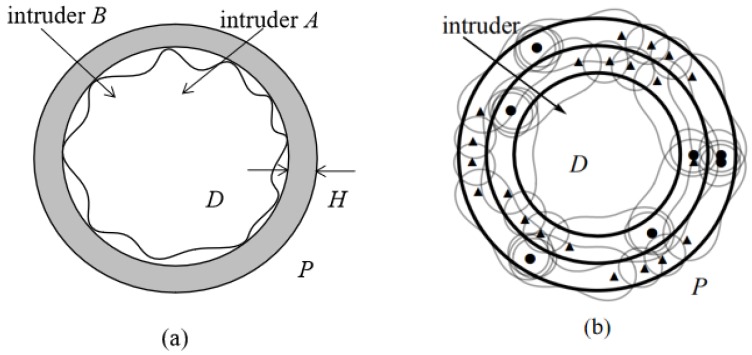
(**a**) The monitoring area *D* and the annulus barrier coverage *P* of width *H*; (**b**) A deployment scheme the monitoring area *D*, the annulus barrier coverage *P*, solid circle represents transmitter and triangle represents receiver.

To improve coverage quality for a particular protected region *D*, we aim to construct a BR circular barrier *P* with the breadth not smaller than a predefined width *H*, while the minimum detection SNR within the circular barrier coverage is not less than a threshold ε. Furthermore, the unit cost of a radar transmitter may be different from a receiver. Then the bistatic radar placement problem is to construct a circular barrier with the minimum total cost of all BR sensors deployed, as shown in Figure 2a. Besides, to satisfy *H* width circular coverage with predefined sensing threshold ε, the circular barrier may be composed of multiple adjacent sub-circles barrier, as shown in Figure 2b. Hence, it is highly non-trivial to optimize the segmentation of the whole circular barrier into multiple adjacent sub-circles barrier and placement of BR transmitters and receivers on the sub-circles.

Usually, the unit cost of transmitter is higher than that of receiver. We quantify their costs using $\lambda =C_{T}/C_{R}>1$, where $C_{T}$ represents the unit cost of transmitter and $C_{R}$ represents the unit cost of receiver. Given that we have *M* transmitters and *N* receivers, the minimum cost coverage problem can be then formulated as follows.

(5) $$\begin{array}{c} \mathrm{minimize}\;MC_{T}+NC_{R}\\ s.t.\;l\left(A\right)\leq l_{\max}^{2}\;\forall A\in P \end{array}$$

Here, $l\left(A\right)\leq l_{\max}^{2}$ is equivalent to $SNR_{A}^{\max}\geq \varepsilon$. We must ensure that the detecting SNR of each point on the BR barrier is greater than or equal to ε.

Given the constraints of BR’s physical parameters and predefined width *H* of circular BR barrier, a single barrier is often insufficient to meet the circular coverage requirements. In belt barrier coverage, Ammari [9] divides the original belt barrier into several identical parallel sub-barriers, and uses the same sensor placement sequence on each sub-barrier. Comparing to the belt barrier, the division problem of circular barrier and BR’s placement problem of multiple sub-circles are more complex to solve. First, the circular barrier may be divided into multiple sub-circles with different width and different length, hence how to divide the circular barrier into multiple sub-circles and how to determine BR’s placement sequence for those sub-circles are non-trival issues. Second, the minimum number of sensors required for each sub-circle may be different, requiring each circle to be handled individually. We demonstrate a possible solution in Figure 2b where circle represents transmitter, triangle represents receiver, and the circular with width *H* is the barrier area we intend to build. We can see that BR sensors are placed with different sequences in different sub-circles, and the number of BR transmitters and receivers in each sub-circle is different.

### 3.3. Basic Deployment Pattern $T_{i}-R^{n}-T_{i+1}$

We consider placing sensors on a single circle. For convenience, we set transmitter $T_{1}$ as the first transmitter with coordinate (r,0). Similar to [7], we use *S* to represent the placement sequence of sensors, for example, $S_{1}≐(T_{1},R_{1},R_{2}$, $T_{2}),S_{2}≐\left(R_{1},T_{1},T_{2},R_{2}\right)$, and etc. For two placement sequences (T,R) and (R,T), they both have the same coverage effect according to the SNR Equation (1) and the characteristics of the Cassini oval line (Figure 1). We hence derive that $\left(T,R^{n}\right)$ and its mirror mode $\left(R,T^{n}\right)$ have the consistent coverage area. Without lossing generality, we focus on the following two placement sequences: $S_{1}=\left(T_{i},R_{1},R_{2},R_{3},\ldots ,R_{n},T_{i+1}\right)$, $S_{2}=\left(R_{i},T_{1},T_{2},T_{3},\ldots ,T_{n},R_{i+1}\right)$, where $T_{i}$ and $T_{i+1}$ represent the *i*th and the i+1th transmitter, respectively, and $R_{i}$, $R_{i+1}$, represent the *i*th and the i+1th receiver. The two sequences have the same coverage effect.

**Figure 3:**
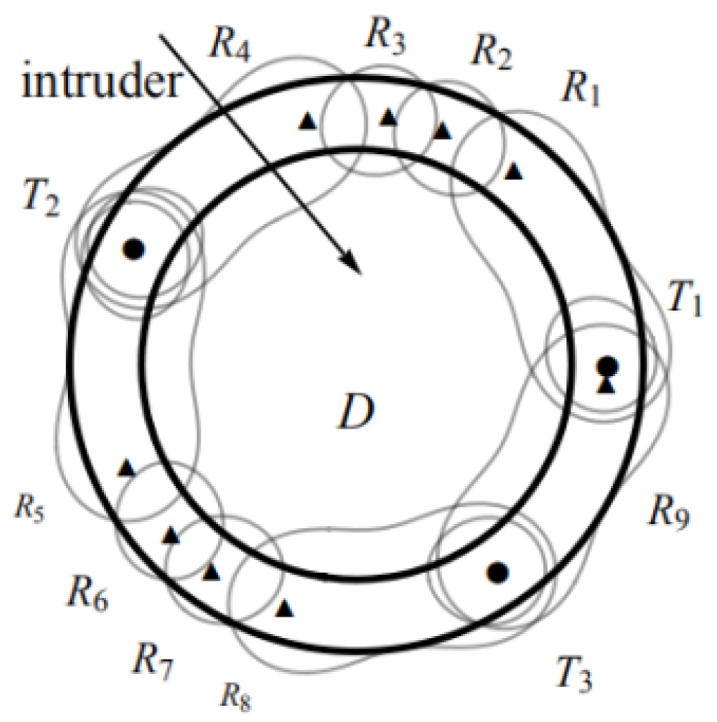
A deployment scheme of circle: The monitoring area *D*, solid circle represents Bistatic Radar (BR) transmitter and triangle represents BR receiver.

However, since the unit cost of transmitter is higher than that of receiver, sequence $S_{1}$ is selected. For convenience, we set the placement sequence as $P_{i}^{n}=\left(T_{i},R^{n},T_{i+1}\right)$, where $T_{i}$ is shared by $P_{i-1}^{n_{1}}$ and $P_{i}^{n_{2}}$. The placement sequence of circular barrier coverage may be composed of several sequences $P_{i}^{n_{j}}$, for example the sequences of sensors $\left(T_{1},R_{1},R_{2},R_{3},R_{4},T_{2}\right)$, $\left(T_{2},R_{5},R_{6},R_{7},R_{8},T_{3}\right)$, and $\left(T_{3},R_{9},T_{1}\right)$, as shown in Figure 3.

Next, in Section 4, we focus on how to solve the barrier placement problem on a single sub-circle barrier of width *h*. Then, in Section 5, we propose two strategies to divide circular barrier into multiples sub-circles and solve the minimum cost placement problem of circular barrier with width *H* and predefined detection threshould.

## 4. Solution for Single Circle with Width *h*

In this section, we focus on the sensor optimized deployment on a single circle with width *h*. We need to use basic deployment pattern $P_{i}^{n}=\left(T_{i},R^{n},T_{i+1}\right)$ to complete the deployment. First, we will consider how to deploy the first pair of sensors $T_{i}-R_{1}$. In Section 4.1, we determine how to choose the most suitable deployment model through detailed analysis. We mainly discuss the waist model (Section 4.1.1) and elliptical model (Section 4.1.2). Then, in Section 4.2.1, we determine the deployment location of other sensors in deployment pattern $P_{i}^{n}$ through detailed mathematical calculation. The effective coverage width (Section 4.2.2) and effective coverage length (Section 4.2.3) of pattern $P_{i}^{n}$ are calculated, and the coverage validity proof of pattern $P_{i}^{n}$ is given (Section 4.2.2). Since a single circle may require multiple patterns $P_{i}^{n}$ to complete deployment, we discuss the combination of multiple patterns $P_{i}^{n}$ in Section 4.3 and give detailed mathematical proof. We know that a single circle may not be deployed in integer pattern $P_{i}^{n}$, so in Section 4.4, we discuss the coverage of the boundary part whose length is less than the coverage length of pattern $P_{i}^{n}$, and give a suitable coverage method through detailed mathematical calculation.

### 4.1. $T_{i}-R_{1}$ Coverage Model

We first determine the coverage model of the first pair of sensors $T_{i}-R_{1}$ in sequence $P_{i}^{n}$. As mentioned in Section 3.1 for several effective sensor coverage patterns, we discuss model (c) and (d) as follows.

#### 4.1.1. Case of Waist

In this case, the sensor’s covecircular area exhibits the shape of a waist, as shown in Figure 1c. An intuition is to place sensors in the middle of the circle so that we can make rational use of the effective coverage area of each sensor, and also facilitate efficient processing and calculation.

We start placing sensors in a counterclockwise direction, i.e., the first transmitter $T_{1}\left(r,0\right)$, followed by the first receiver, and etc.

**Theorem** **1.** *For a pair of sensors T−R, the vulnerability in the two points where the midnormal of line segment $\bar{TR}$ intersects with the covecircular area is the largest, and it is also the place where the signal-to-noise ratio is the smallest.*

**Proof** **of** **Theorem** **1.** The proof of Theorem 1 is given in the Appendix A. □

**Figure 4:**
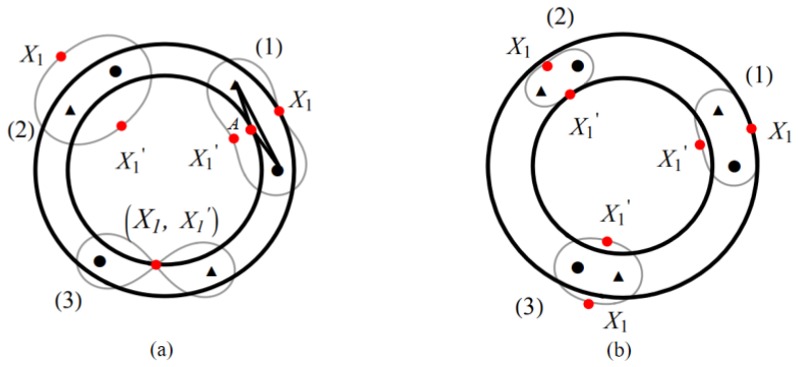
Solid circle represents BR transmitter and triangle represents BR receiver. (**a**) The sensor location schematic diagram (waist); (**b**) The sensor location schematic diagram (ellipse).

We denote the two points in Theorem 1 as $X_{1}$ and ${X_{1}}^{\prime }$, respectively. In Figure 4a, we only need to consider how to determine the location of these two points $X_{1}$, ${X_{1}}^{\prime }$. We divide the problem into three cases as follows.

1.1 Suppose that there is only one point between $X_{1}$ and ${X_{1}}^{\prime }$ at the inner and outer boundaries of the annulus, and we find that point $X_{1}$ at the outer boundary is better than point ${X_{1}}^{\prime }$ at the inner boundary, as shown in Figure 4a(1). Since point ${X_{1}}^{\prime }$ is on the inner boundary, T−R cannot meet the coverage requirement, one of the cases is shown in Figure 4a(3).
1.2 Suppose that the two points $X_{1}$ and ${X_{1}}^{\prime }$ are not inside and outside the boundary of the annulus. To meet the coverage requirements, $X_{1}$ must be outside of the outer boundary and ${X_{1}}^{\prime }$ must be inside of the inner boundary, as shown in Figure 4a(2). However, it draws closer to the distance between T−R, compared to the first case, the length of the cover is smaller.
1.3 Suppose that the two points $X_{1}$ and ${X_{1}}^{\prime }$ are in the inner and outer boundaries of the annulus separately, as shown in Figure 4a(1). In this case, ${X_{1}}^{\prime }$ is on the location of point *A*, but $X_{1}$ cannot be on the outer boundary of the annulus, hence it is not feasible.

Based on the above three cases, we conclude that the best solution is that point $X_{1}$ is on the outer boundary of the annulus, and point ${X_{1}}^{\prime }$ is inside the inner boundary of the annulus to meet the coverage requirements.

#### 4.1.2. Case of Ellipse

The coverage area of the sensors is oval in this case, as shown in Figure 1d. We also denote two points as $X_{1}$ and ${X_{1}}^{\prime }$. Similarly, we discuss three cases as follows.

2.1 Suppose that there is only one point between $X_{1}$ and ${X_{1}}^{\prime }$ inside and outside the boundary of the annulus. We find that when point $X_{1}$ on the outer boundary of the annulus, the covering area is tangent to the outer boundary of the annulus at point $X_{1}$. It is obvious that it does not meet the coverage requirements, as shown in Figure 4b(1), and the same conclusion can be drawn when point ${X_{1}}^{\prime }$ is on the inner boundary, as shown in Figure 4b(2).
2.2 Assuming that the two points $X_{1}$ and ${X_{1}}^{\prime }$ are not inside and outside the boundary of the annulus, it is possible to satisfy the coverage condition at this point, but the coverage length is shorter than that of the waist model, as shown in Figure 4b(3).
2.3 Suppose that both points $X_{1}$ and ${X_{1}}^{\prime }$ are inside and outside the boundary of the annulus, similar to the case of the waist coverage. This case is not feasible.

To conclude the above cases we have discussed, we will focus on the waist coverage in the remaining of the paper.

### 4.2. Placement Pattern of $P_{i}^{n}=\left(T_{i},R^{n},T_{i+1}\right)$

In this section, we present the deployment scheme for sequence $P_{i}^{n}$, including determining the specific location of transmitters and receivers, proposing the method to compute the effective coverage width and length, and also providing the proof of the coverage reliability of sequence $P_{i}^{n}$. Here, the coverage area formed by the second receiver $R_{2}$ and transmitter $T_{i}$ may be a waist region or two separate regions, as shown in Figure 1a,c. For convenience, we choose the latter to discuss.

#### 4.2.1. Determining Sensor Locations

**Figure 5:**
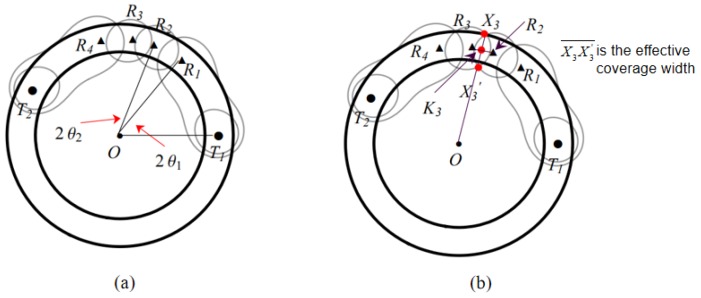
The deployment scheme of $T_{1}-R^{4}-T_{2}$: (**a**) The center angle; (**b**) The effective coverage width.

Given sequence $P_{i}^{n}=\left(T_{i},R_{1},R_{2},\ldots ,R_{n},T_{i+1}\right)$, the arc is divided into several successive sub-arcs. We aim to determine the location of the radar sensors to cover the entire circular portion. We first introduce the concept of the central angle. For the arc of sequence $P_{i}^{n}$, we use $\left(2\theta_{1},2\theta_{2},\ldots ,2\theta_{n+1}\right)$ to represent the sequence of its central angle, where $\theta_{1}=\frac{1}{2}∠T_{i}OR_{1}$, $\theta_{2}=\frac{1}{2}∠R_{1}OR_{2}$, …, $\theta_{n}=\frac{1}{2}∠R_{n-1}OR_{n}$, $\theta_{n+1}=\frac{1}{2}∠R_{n}OT_{i+1}$, as shown in Figure 5a. To determine the location of the corresponding sensors, we need to get the values of these central angles. We compute the central angles based on Theorem 2.

**Theorem** **2.** *For a sequence $P_{i}^{n}$, the central angles can be calculated as follows: (1) If n is odd, then $\theta_{1}=\theta_{n+1}$, $\theta_{2}=\theta_{n}$, …, $\theta_{\frac{n+1}{2}}=\theta_{\frac{n+1}{2}+1}$, (2) if n is even, then $\theta {}_{1}=\theta_{n+1}$, $\theta_{2}=\theta_{n}$, …, $\theta_{\frac{n}{2}}=\theta_{\frac{n}{2}+2}$, $\theta_{\frac{n}{2}+1}=\theta_{\frac{n}{2}+1}$, where $\theta_{x}$ is the central angle, $x=1,2,\ldots ,\left\lceil \frac{n+1}{2}\right\rceil$, r is the length between the central position of the part of annulus and the center of the circle, $R_{\max}$ is the length between the outer boundary of the annulus and the center of the circle.*

(6) $$\theta_{x}=\left\{\begin{array}{c} \arccos\frac{r^{2}+R_{\max}^{2}-l_{\max}^{2}}{2rR_{\max}},\;\;x=1\\ \arccos\left(\frac{(R_{\max}^{2}+r^{2})\cos\sum_{i=1}^{x-1}\theta_{i}-\sqrt{{\left(R_{\max}+r\right)}^{2}{\left(R_{\max}-r\right)}^{2}(\cos^{2}\sum_{i=1}^{x-1}\theta_{i}-1)+l_{\max}^{4}}}{2rR_{\max}}\right)-\sum_{i=1}^{x-1}\theta_{i},\;x>1 \end{array}\right.$$

**Proof** **of** **Theorem** **2.** The proof of Theorem 2 is given in the Appendix A. □

For the upper and lower bounds of *n* in Theorem 2, it is difficult to give the maximum value $n_{\max}$ of *n* explicitly. We first determine the physical properties of the sensors and the inner and outer radius of the annulus are determined so that we can obtain the maximum central angle of sequence $P_{i}^{n}$. We then derive the maximum value of *n* under this condition, which is the maximum number of receivers in a single sequence $P_{i}^{n}$. Theorem 3 gives the computing equation.

**Theorem** **3.** *For a sequence $P_{i}^{n}$, the largest circle angle can be covered by it is $2\theta_{\max}$, the maximum number of receivers can be derived by the following equation: $\sum_{i=1}^{n_{\max}+1}2\theta_{i}=2\theta_{\max}$, where $\theta_{\max}$ can be calculated as follows.*

(7) $$\theta_{\max}=\arccos\left(\frac{\left(R_{\max}^{2}+r^{2}\right)\cdot {\left(R_{\max}-r\right)}^{2}-l_{\max}^{4}}{2rR_{\max}\cdot {\left(R_{\max}-r\right)}^{2}}\right)$$

**Proof** **of** **Theorem** **3.** The proof of Theorem 3 is given in the Appendix A. □

#### 4.2.2. Effective Coverage Width of $P_{i}^{n}=\left(T_{i},R^{n},T_{i+1}\right)$ and Coverage Quality

We now discuss the coverage quality of sequence $P_{i}^{n}$, i.e., whether the minimum width of the coverage area is greater than the width of the circle.

We first discuss the effective coverage width of sequence $P_{i}^{n}$. In $P_{i}^{n}$, the coverage area of each pair of sensors T−R is different. With the number of receivers increased, the width of the receiver which is farther away from the transmitter is smaller. Therefore, we only need to find out the minimum coverage width of sequence $P_{i}^{n}$ when the number of receivers is determined, that is also the coverage width of the receiver in the middle of sequence $P_{i}^{n}$, i.e., the effective coverage width of sequence $P_{i}^{n}$.

As shown in Figure 5b, the value of *n* is four, the cover sequence is $P_{i}^{4}$. We observe that the middle position of sequence is where the line segment $\bar{X_{3}{X_{3}}^{\prime }}$ is in, and the width of the coverage is the length of line segment $\bar{X_{3}{X_{3}}^{\prime }}$. It is obvious that the actual width of the coverage is greater than the width of the annulus, and this is in fact the effective coverage width.

For sequence $P_{i}^{n}$, the effective coverage width is the length of line segment $\bar{X_{mid}{X_{mid}}^{\prime }}$, but the calculation of this value is very complicated. For convenience, to meet the coverage requirements, we introduce the concept of two times value. Assuming that the intersection point between line segment $\bar{X_{mid}{X_{mid}}^{\prime }}$ and the two nearest receivers is $K_{mid}$, we use $2∥X_{mid}K_{mid}∥$ as an approximate value for the length of line segment $\bar{X_{mid}{X_{mid}}^{\prime }}$. For example, in Figure 5b, we use $2∥X_{3}K_{3}∥$ as an approximate value for the length of line segment $\bar{X_{3}{X_{3}}^{\prime }}$. Please note that an analysis of the approximate method is given in the Appendix A. We hence obtain the equation to compute the effective coverage width of sequence $P_{i}^{n}$, which is given in Theorem 4.

**Theorem** **4.** *For sequence $P_{i}^{n}$, to meet the coverage requirements, when n takes different values, the effective coverage area width is given as follows.*

(8) $$d_{n}=2\left(R_{\max}-r\cdot \cos\theta_{\frac{n}{2}+1}\right),1\leq n\leq n_{\max}$$

**Proof** **of** **Theorem** **4.** The proof of Theorem 4 is given in Appendix A. □

We have now obtained the effective coverage width of sequence $P_{i}^{n}$. To verify the reliability of the sequence coverage, we only need to compare the effective coverage width with the width of the circle, which is described in Theorem 5.

**Theorem** **5.** *In sequence $P_{i}^{n}$, until the number of receivers n is taken to the upper bound, $P_{i}^{n}$ always meets the requirements of coverage, i.e., the coverage of sequence $P_{i}^{n}$ is reliable.*

**Proof** **of** **Theorem** **5.** The proof of Theorem 5 is given in Appendix A. □

#### 4.2.3. Effective Coverage Length of $P_{i}^{n}=\left(T_{i},R^{n},T_{i+1}\right)$

In Section 4.2.1 and Section 4.2.2, we give the location of sensors in sequence $P_{i}^{n}$, the method to compute the effective coverage width, and provide the proof of the sequence coverage quality. We now determine the effective coverage length of sequence $P_{i}^{n}$ when the number of receivers *n* is determined.

**Figure 6:**
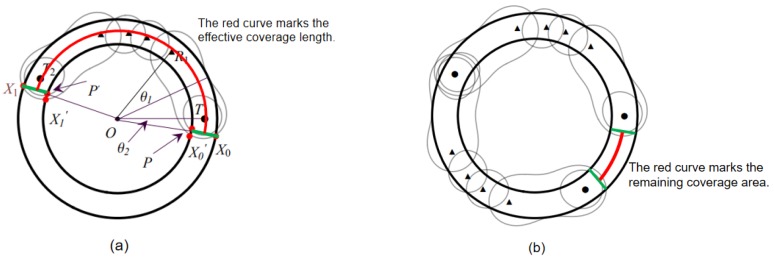
(**a**) The effective coverage length; (**b**) The overlay boundary.

We leverage the central angle to solve this problem. As shown in Figure 6a, we observe a pair of sensors $T_{1}-R_{1}$ intersect the circle at two points $X_{0}$, ${X_{0}}^{\prime }$. We need the line segments $\bar{X_{0}{X_{0}}^{\prime }}$ as the starting position to compute the effective coverage length, but this computation is very cumbersome, requiring simplifying. We consider that in real applications, it requires multiple sequences to complete the deployment of the barrier, and multiple sequences will be end to end. In Figure 6a, the coverage area of the last sequence at least needs to cross point $X_{0}$, existing coverage areas overlap. We connect point $X_{0}$ to the center *O*, and it will intersect the circular at point $X_{0}$ and point *P*. Because the line segment $\bar{X_{0}P}$ is still in the coverage of the sensors, we can consider line segment $\bar{X_{0}P}$ as the starting position to compute the effective coverage length. By symmetry, we find that $\theta =\theta_{2}$. Since the other angle has been given by the equation in Theorem 2, we can obtain the effective coverage length of sequences $P_{i}^{n}$ by using the above conditions. Theorem 6 gives the computing equation.

**Theorem** **6.** *For sequence $P_{i}^{n}$, when the number of receivers n takes different values, the length of the effective coverage area is calculated as follows, where $n=1,2,\ldots ,n_{\max}$, $n_{\max}$ represents the maximum number of receivers, hence meeting the coverage conditions.*

(9) $$L_{n}=\left\{\begin{array}{c} 2r\cdot (2\sum_{i=1}^{\frac{n+1}{2}}\theta_{i}+\theta_{2}),\;\;\;\;n\;\mathrm{is}\;\mathrm{odd}\\ 2r\cdot (2\sum_{i=1}^{\frac{n}{2}}\theta_{i}+\theta_{\frac{n}{2}+1}+\theta_{2}),\;n\;\mathrm{is}\;\mathrm{even} \end{array}\right.$$

**Proof** **of** **Theorem** **6.** The proof of Theorem 6 is given in Appendix A. □

### 4.3. Optimal Placement Sequence on Single Circle with Width h

We have so far solved the deployment of a single sequence $P_{i}^{n_{j}}$. We know that a circle may have multiple $P_{i}^{n_{j}}$ sequences. We use sequence $Y=(n_{1},n_{2},\ldots ,n_{t})$ to represent the coverage sequence on a single circle, denoted as $\left(P_{i}^{n_{1}},P_{i+1}^{n_{2}},\ldots ,P_{t-1}^{n_{t}}\right)$. To addess this problem, we first need to find out the numerical relation between $n_{1},n_{2},\ldots ,n_{t}$ in order to determine the optimal placement sequence. We formally formulate this problem as follows. Suppose we have $M_{1}$ transmitters and $N_{1}$ receivers, and we use sequence $Y=(n_{1},n_{2},\ldots ,n_{t})$ to form a barrier with width *h*, how do we maximize the length of this barrier?

Let us consider the general sequence $S=(T_{1},R^{n_{1}},T_{2},R^{n_{2}},T_{3})$, where $n_{1}$, $n_{2}$ is the number of receivers. Assume that $n_{1}$ is odd, $n_{2}$ is even, and $n_{1}<n_{2}$. We can construct another sequence, it is $S^{\prime }=\left(T_{1},R^{\frac{n_{1}+n_{2}-1}{2}},T_{2},R^{\frac{n_{1}+n_{2}+1}{2}},T_{3}\right)$, where $\frac{n_{1}+n_{2}-1}{2}$ and $\frac{n_{1}+n_{2}+1}{2}$ are odd or even. We define $\psi_{1}$, $\psi_{2}$ represent the coverage angle of sequence *S* and $S^{\prime }$, respectively, we can compare the coverage length of the two sequence by comparing the value of the two parameters. We now have Theorem 7.

**Theorem** **7.** *For sequences S and $S^{\prime }$, assuming that $n_{1}$ is odd, $n_{2}$ is even, and $n_{1}<n_{2}$, we have (1) If $n_{2}-n_{1}=1$, then $\psi_{1}=\psi_{2}$; (2) If $n_{2}-n_{1}>1$, then $\psi_{1}<\psi_{2}$.*

**Proof** **of** **Theorem** **7.** The proof of Theorem 7 is given in the Appendix A. □

When both $n_{1}$ and $n_{2}$ are even or odd, we can also use the proof of Theorem 7 to prove: If $\left|n_{1}-n_{2}\right|\leq 2$, then $\psi_{1}=\psi_{2}$; if $\left|n_{1}-n_{2}\right|>2$, then $\psi_{1}<\psi_{2}$. Therefore, we find that with $M_{1}$ transmitters and $N_{1}$ receivers, without considering the boundary problem, we can get the longest coverage barrier by using multiple same deployment sequences, and this deployment sequence is also known as the optimal deployment sequence. We therefore use this optimal deployment sequence in this paper.

### 4.4. Overlay Boundary Problem on Single Circle with Width h

In this section, we prove that the same sequence should be used as much as possible to complete the deployment. We first determine how many identical sequences are needed to complete the deployment and deal with the problem of the overlay boundary, as shown in Figure 6b.

Suppose the deployment sequence of a circle is S=A+B, where $A=xP_{i}^{n}$, representing *x* sequence $P_{i}^{n}$, and *B* is the remainder. We use *C* to represent the coverage angle of a single sequence $P_{i}^{n}$, use *D* to represent the angle of the overlapping part, and use Lt to represent the angle of the remainder. We can get $C=L_{n}/r$, Lt=2π−x·C+D, $D=2\left(x-1\right)\cdot \theta_{2}$. Because of Lt≤C, it is only necessary to place the appropriate receivers in the remaining part. But we cannot directly compare Lt=2π−x·C+D with the angle of sequence $P_{i}^{n}$ to determine the number of receivers due to the overlap of *x* optimal sequences, for example, if $-2\theta_{2}<Lt<0$, it still satisfies the coverage requirements. We hence get a reasonable range of the remaining angle, i.e., $-2\theta_{2}\leq Lt\leq 2a_{n}-2\theta_{2}$, where $a_{n}$ is given in Theorem 8.

**Theorem** **8.** *For the circle which is covered by sequence $P_{i}^{n}$, the number of receivers needed for the remaining arcs is computed as follows: (1) If $-2\theta_{2}\leq Lt\leq 0$, there is no need for additional receivers; (2) if $0<Lt\leq 2a_{n}-2\theta_{2}$, the number of receivers is determined by the following equation: if $2a_{k-1}<Lt+2\theta_{2}\leq 2a_{k}$, then it needs* k *receivers, where 1≤k≤n, Lt represents the angle of the remaining part of arc, and $a_{k}$ can be calculated as follows.*

(10) $$a_{k}=\left\{\begin{array}{c} 0,\;\;\;\;\;\;k=0\\ 2\sum_{i=1}^{\frac{k+1}{2}}\theta_{i},\;\;\;\;k\;\mathrm{is}\;\mathrm{odd}\\ 2\sum_{i=1}^{\frac{k}{2}}\theta_{i}+\theta_{\frac{k}{2}+1},\;k\;\mathrm{is}\;\mathrm{even}\;\mathrm{and}\;k\neq 0 \end{array}\right.$$

**Proof** **of** **Theorem** **8.** The proof of Theorem 8 is given in Appendix A. □

Up to this point, we have solved the problem of BR sensor deployment on a single circle. In the next section, we will discuss the minimum coverage cost of the whole circular barrier coverage with width *H* (H>h).

## 5. Solution of Minimum Coverage Cost on the Whole Circular Barrier with Width *H*

We now discuss how to solve the minimum cost placement problem of BR sensors for the whole circular barrier coverage. Because the coverage area of bistatic radar sensors is variable and one transmitter (receiver) can form sensor pairs with multiple receivers (transmitters), it is very difficult to obtain the optimal solution of the minimum cost coverage problem. Therefore, we consider the approximate optimal solution of this problem. In Section 4, we discuss the optimal deployment method of a single circle with width *h*. On this basis, we discuss the approximate optimal deployment method of the whole circular barrier with width H(H≫h). Since the width *H* of whole circular barrier exceeds the coverage width of single BR circle barrier, we need multiple adjacent sub-circle barrier to cover the whole circular areas in a cooperative way.

Consider that the width of the waist coverage model of BR sensor is variable, and each sub-circle of the circular barrier coverage may have different coverage length and width, and different BRs’ placement sequence. Hence we need to further investigate the optimal sub-circle’s division and BR placement algorithm to achieve minimum cost placement of BR sensors on the whole circular barrier with predefined coverage quality. We first design a width equalization division strategy and BR placement algorithm for circular barrier. We analyze and validate effectiveness of the width equalization division strategy and algorithm by simulation experiments. Secondly, by analyzing the results of the experiment, we further propose an adaptive division strategy and approximate optimization BR placement algorithm for circular barrier. By comparing with the width equalization strategy and algorithm in simulation experiments, we validate the effectiveness of the adaptive division strategy and minimum cost placement BR deployment algorithm on the whole circular barrier with Width *H* and required coverage quality.

In this paper, we mainly consider the calculation of the coverage cost of the sensors when $C_{T}>C_{R}$, where $C_{T}$ represents the unit cost of transmitter and $C_{R}$ represents the unit cost of receiver.

### 5.1. Equal Circle width Division Strategy and BR Sensors Placement Algorithm

#### 5.1.1. Equal width Circle Division and Optimal Placement Algorithm

**Figure 7:**
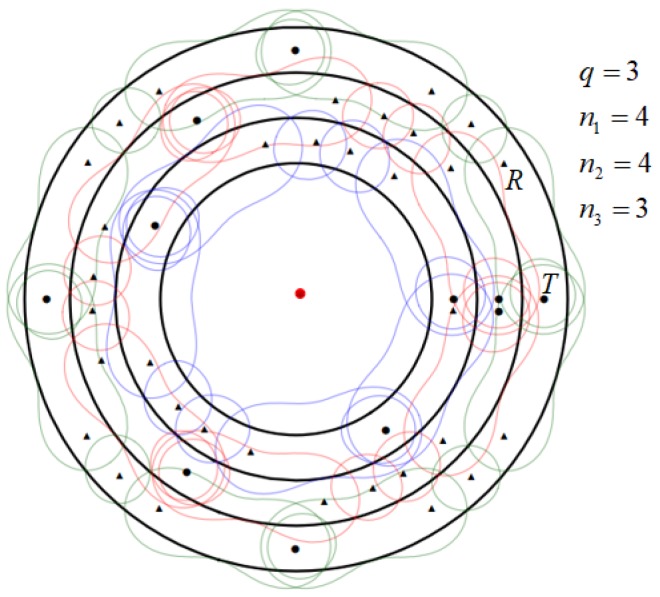
The schematic diagram of the width equalization strategy: (1) The barrier consists of three sub-barriers; (2) The deployment patterns of each sub-barrier are $T_{i}-R^{4}-T_{i+1}$, $T_{i}-R^{4}-T_{i+1}$ and $T_{i}-R^{3}-T_{i+1}$, respectively; (3) Solid circle represents BR transmitter and triangle represents BR receiver.

For ease of understanding, we give a detailed pattern diagram, as shown in Figure 7. This strategy divides the circular barrier into *q* circles, and each of them has the same width. Due to different radius of each circle, we cannot use the same deployment sequence for all the circles. We first divide each circle separately, and apply the same deployment idea to analyze and calculate the specific radius of the circle to get the minimum coverage cost of each circle. We finally obtain the total cost of coverage with all the costs.

We need to determine the number of circles, i.e., *q*, and obtain value *n* in sequence $P_{i}^{n}$, which is the number of receivers. For the two parameters *q* and *n*, if the two parameters have a certain relationship, we can easliy use one of them to determine the other. However, we find that the two parameters are not related. Therefore, for the number of circle *q* and *n*, we design an exhaustive algorithm to enumerate *q* and *n*. By comparison, we can get the minimum coverage cost under meeting the conditions.

We find that the width of the circle exists the upper and lower bounds, so the number of circles also has a certain range. We mainly use the waist shape covering model to maintain the waist shape of the sensor, and we need to satisfy the following conditions: $\sqrt{2}l_{\max}<d\left(T,R\right)<2l_{\max}$, then we get the constraint condition of the circle’s width is $0<h<\sqrt{2}l_{\max}$. For the number of circles *q*, we can first determine $\frac{H}{\sqrt{2}l_{\max}}<q$. For the upper bound of *q*, in practice, we find that when *h* is reduced to a certain width while *q* is taken to an upper bound. Then, with the number of circles *q* increases, the optimal number of sensors continues to increase, resulting in cost increase. We set up this upper bound as μ, so there is $\frac{H}{\sqrt{2}l_{\max}}<q<\mu$. After determining the constraints of the two parameters, the exhaustive algorithm can be used to calculate the minimum coverage cost.

We increase the number of circles from $q=\left\lceil \frac{H}{\sqrt{2}l_{\max}}\right\rceil$ to q=μ−1, each time by 1 unit. For each circle, we enumerate *n* and calculate the minimum coverage cost on each circle, then sum up them to get the global minimum coverage cost. Finally, we compare the minimum coverage cost in all cases so as to get the optimal solution. Here, we set the global minimum coverage cost as $C_{total}^{*}$, the optimal number of circles as $q^{*}$. The specific calculation process is given in Algorithms 1 and 2, respectively.

**Algorithm 1** Find $q^{*}$ and $C_{total}^{*}$ When $C_{T}>C_{R}$
 1: Initialization: σ=φ, τ=φ, υ=φ, $C_{total}=0$ 2: **for**
$q\leftarrow \left\lceil \frac{H}{\sqrt{2}l_{\max}}\right\rceil :\mu$
**do** 3: **for**
t←1:q
**do** 4:  **for**
$n\leftarrow 1:Q_{t}$
**do** 5:   h←H/q 6:   $C_{n}\leftarrow Compute\left(n,h\right)$ 7:   $\sigma \leftarrow \sigma \cup \left\{C_{n}\right\}$ 8:  **end for** 9:  $C_{total}\leftarrow C_{total}+\min\left(\sigma \right)$ 10:  σ.clear() 11: **end for** 12: $\tau \leftarrow \tau \cup \left\{q\right\}$ 13: $\upsilon \leftarrow \upsilon \cup \left\{C_{total}\right\}$ 14: $C_{total}\leftarrow 0$ 15: **end for** 16: $C_{total}^{*}\leftarrow \min\left(\upsilon \right)$ 17: $index\leftarrow \upsilon .index(C_{total}^{*})$ 18: $q^{*}\leftarrow \tau \left(index\right)$

**Algorithm 2**Compute(n,h) Compute Cost When *n* and *h* are determined 1: Initialization:M=0,N=0,RT=0,RR=0,Lt=0,Cost=0 2: **for**
$x\leftarrow 1:3\cdot \frac{2\pi }{C}$
**do** 3: Lt←2π−x·C+D 4: **if**
$-2\theta_{2}\leq Lt\leq 2a_{n}-2\theta_{2}$
**then** 5:  Compute *M* and *N*, Compute *RT* and *RR* 6:  M←M+RT, N←N+RR 7:  break 8: **end if** 9: **end for** 10: $Cost=C_{T}\cdot M+C_{R}\cdot N$ 11: return Cost

#### 5.1.2. Experimental Result of the Equal Circle Width Division Strategy

In the simulation experiments, we set up the parameters of BR sensor network, as show in Table 2. $l_{\max}^{2}$ denotes the detectability threshold of BR sensors, and *h* denotes the width range of sub-barrier. *q* denotes the number of sub-barriers, which is determined by the perimeter barrier width *H* and the maximum coverage width of the single pair BR sensor. $R_{m}$ denotes the minimum inner radius of the perimeter barrier. Then $C_{T}$ denotes the unit price of the transmitter and $C_{R}$ denotes the price of the receiver. And λ denotes the SNR threshold of the perimeter barrier. The simulation experiments are performed on 64-bit Windows 10 system; Programming language is C++ and Python. The algorithms are realized in C++ language. And the deployment visualization of deployment results is realized in Python language.

Firstly, we consider the influence of the annulus barrier width *H* on the optimal number of circles $q^{*}$, the optimal circle width *h*, and the minimum coverage cost Cost. We find that with the increase of *H*, the larger the λ, the greater the $q^{*}$ will be. The bigger the λ, the smaller the Cost will be. Then the bigger the λ, the smaller the *h* will be.

**Figure 8:**
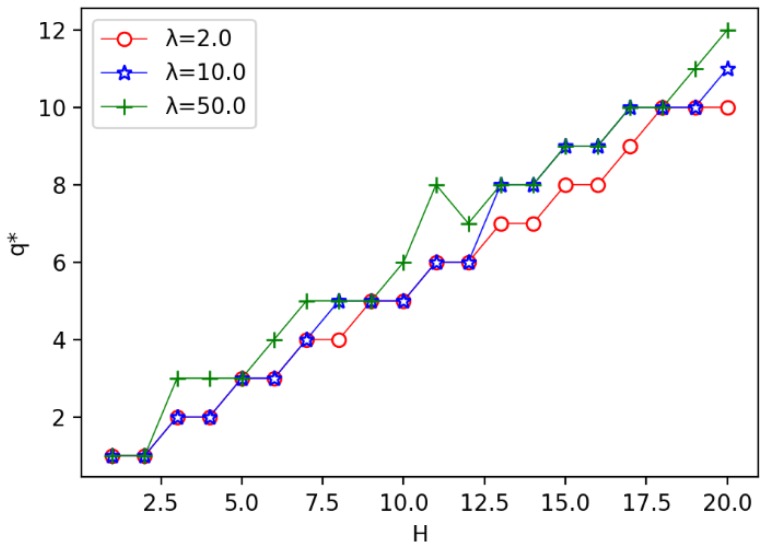
The influence of width *H* on the number of optimal circles $q^{*}$.

We enumerate the width of the annulus barrier from 1 to 20, increasing the width of one unit at a time, to observe the impact process respectively. As shown in Figure 8, we can find that the number of optimal circles $q^{*}$ increases with width *H*. In the Figure 8, we give three different unit costs of a sensor, where $\lambda =\frac{C_{T}}{C_{R}}$, we set λ=2,10,50, respectively. It is observed that when H≤12, the curve of λ=2.0 is almost the same as the curve of λ=10.0. But when λ=50.0, the value of $q^{*}$ is more than or equal to the other two. When 13≤H≤18, the curve of λ=10.0 is almost the same as the curve of λ=50.0. But when λ=2.0, the value of $q^{*}$ is less than or equal to the other two. This explains that under the same width *H*, when ratio λ increases, the number of optimal circles also increases. This is because the increase of unit cost λ means that receiver becomes cheaper, resulting the number of sensors increases and the circle’s width becomes smaller.

We now evaluate the impact of the annulus barrier width *H* on the minimum cover cost.

**Figure 9:**
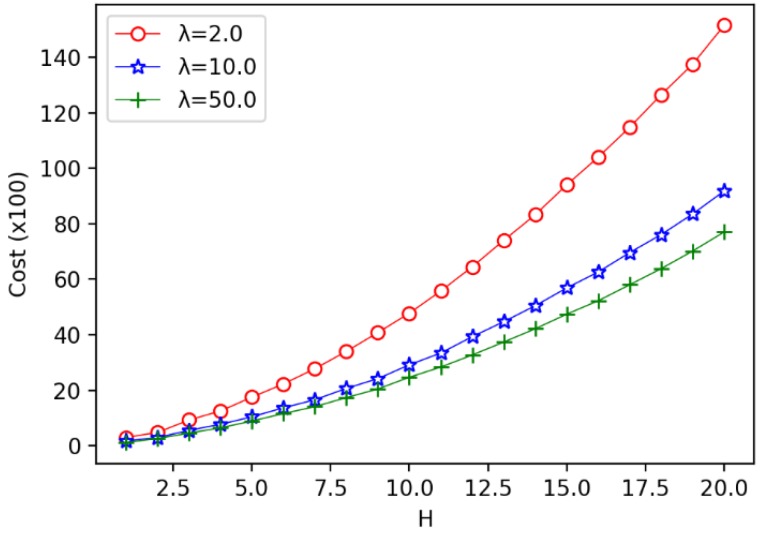
The influence of width *H* on the minimum coverage cost.

As shown in Figure 9. We observe that when H≤4, for the unit price ratio of different sensors, there is little difference in the cost required to cover. But when H≥5, with the increase of the unit price ratio, the difference of coverage cost is becoming more and more obvious. We observe that when the width of annulus barrier is determined, a larger unit cost ratio λ of a sensor implies a cheaper coverage cost. This can be explained by the fact that the larger λ is used, the smaller the unit cost of receiver is required. As a result, we have more number of receivers and less number of transmitters required, and hence the overall coverage cost is reduced. From this result, we conclude that under certain conditions, choosing a larger cost ratio λ to cover the barrier can save the coverage cost.

**Figure 10:**
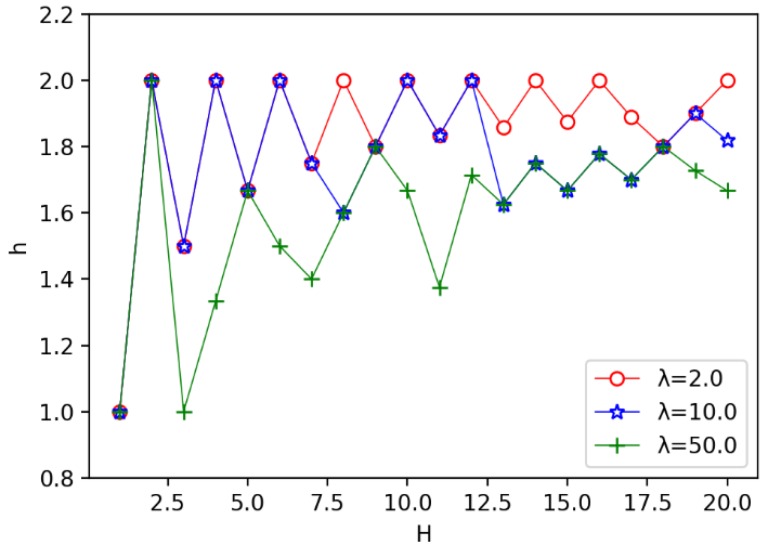
The influence of width *H* on the optimal width *h* of circle.

We also evaluate the impact of the annulus barrier width *H* on the width *h* of the circle. As shown in Figure 10, we observe that for a single function curve which the value of λ is detemined, the value of *h* appears to fluctuate up and down, for example, when λ=50.0, as shown in the green curve in the picture, *h* fluctuates between [1.0,2.0], but with the increase of the annulus barrier width *H*, the function value tends to converge to a fixed value and stables near 1.7. At the same time, we found that with different values of λ, the three function curves are similar, and *h* gradually stabilizes in the range of [1.7,1.9]. We also observe that the larger the λ is used, the smaller the *h* is obtained. Because the receiver is cheap, the amount of the receiver is much more, which makes the width *h* of the circle smaller, which is consistent with the performance in Figure 8.

**Figure 11:**
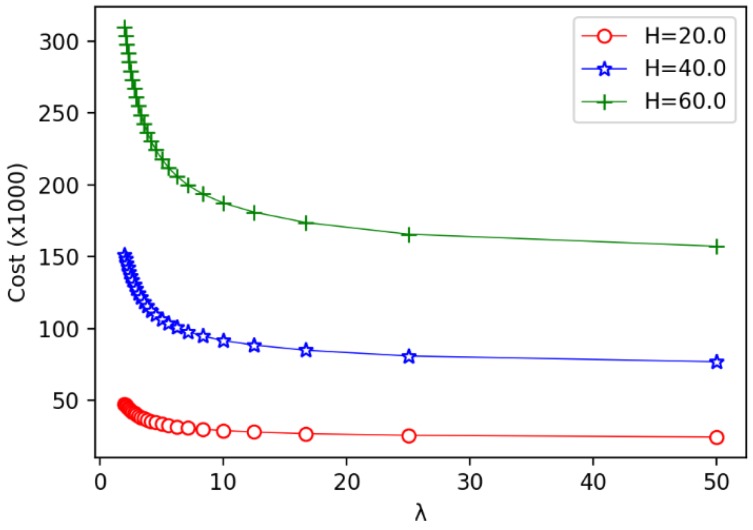
The relationship between the unit price ratio λ and the minimum coverage cost.

**Figure 12:**
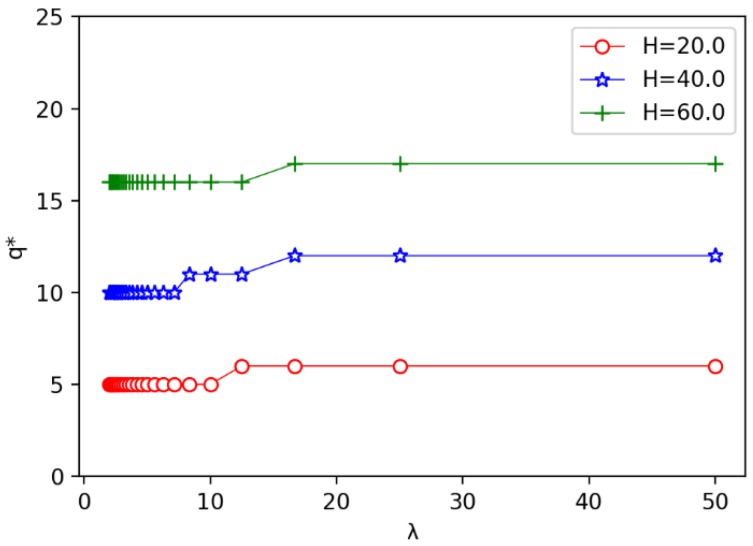
The relationship between the unit price ratio λ and the number of optimal circle $q^{*}$.

**Figure 13:**
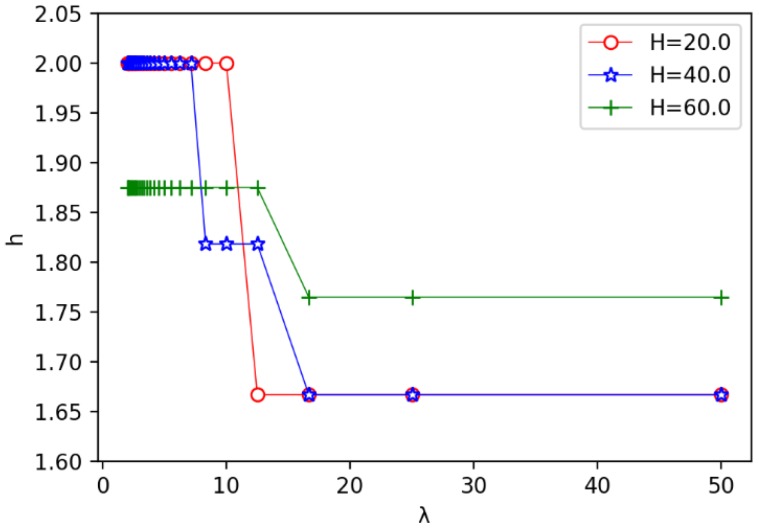
The relationship between the unit price ratio λ and the optimal width *h* of circle.

Secondly, we evaluate the impact of the unit cost ratio λ on other parameters. The results are shown in Figure 11, Figure 12 and Figure 13. In Figure 11, we observe that the speed of the minimum coverage cost decreases the fastest with the unit cost ratio λ between 0 and 10. The greater the annulus barrier width results in less effective coverage costs. From Figure 12, we observe that when the annulus barrier width is different, the number of optimal circles $q^{*}$ increases gradually and tends to be stable as the unit cost ratio λ increases. In addition, the greater the width *H* of the barrier, the greater the value $q^{*}$. In Figure 13, we find that as the unit price ratio λ increases, the value *h* decreases gradually, and also tends to a stable value. At the same time, the larger the width *H* of the barrier, the greater the width of the corresponding optimal circle.

#### 5.1.3. Algorithm Analysis of the Equal Circle Width Division Strategy

In order to determine the approximate degree of the algorithm, we need to determine the lower bound of the theoretical optimal solution $N^{*}$ and the upper bound of the algorithm’s solution *N*. But through specific analysis, we find it difficult to prove.

When the unit price ratio λ and the barrier width *H* are determined, we need to find the cheapest set of sensors that meet the coverage conditions. In order to find the lower bounds of the theoretical optimal solution, we assume that the sensors’ coverage area does not overlap, but because of the following problems, it is difficult to determine the lower bounds. First, the coverage area of the BR sensor is determined by the transmitter and receiver. Secondly, the size of the coverage area is affected by the distance between the sensors. When the distance between the sensors is different, the size of the coverage area is different. Therefore, it is difficult for us to determine the distance between sensors and how many transmitters and receivers are needed, so it is difficult to calculate the price and determine the lower bound of $N^{*}$.

For the upper bound of the algorithm’s solution, we need to consider the worst case. But in this paper, when the unit price ratio λ and the barrier width *H* are determined, the only minimum cost can be obtained according to the algorithm proposed by us. Therefore, there is no worst-case statement.

At the same time, we have given other barrier coverage strategies in the following section, and have carried out detailed experiments to compare, proving that there is a better solution.

### 5.2. Adaptive Circle Width Division Strategy and BR Sensors Placement Algorithm

By observing the experimental results in the width equalization strategy, we find that given the sensor parameters and SNR thresholds, with the increase in the width *H* of the annulus barrier, the optimal width *h* of the circle will gradually stabilize in the range of [1.7,1.9] for different cost ratio λ. Therefore, we choose the width of the circle in this interval, then define a new barrier coverage strategy: For an annulus barrier with width *H*, the barrier can be segmented with $h_{x}\left(1.7\leq h_{x}\leq 1.9\right)$ as the division unit. If *H* is a multiplier of $h_{x}$, it is exactly divided; Otherwise, a circle with the width less than $h_{x}$ is left. We also follow the same experimental environment and calculation methods.

### 5.3. Experiment Result Comparison of Equal Division vs. Adaptive Division Strategy

**Figure 14:**
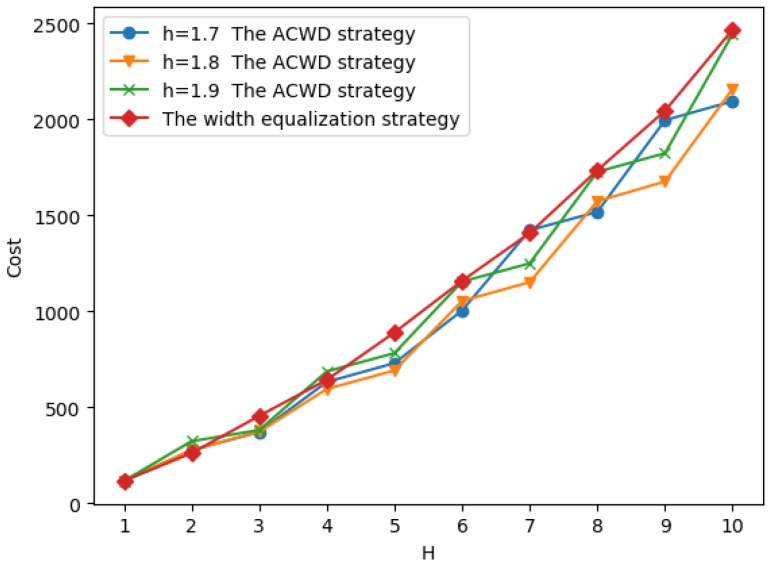
Comparison when 1≤H≤10.

The experimental results of the two strategies mentioned above are shown in Figure 14. At this time, the unit price ratio is λ=CTCTCRCR = 50.

We know that the adaptive placement strategy is from the analysis of experimental results on the equipartition placement strategy. In the simulation experiment of the equipartition placement strategy, we find that when the sensor parameters and the SNR threshold are determined, the optimal width *h* of circle is in the interval [1.7,1.9]. In the adaptive placement strategy, we use $h_{x}\left(1.7\leq h_{x}\leq 1.9\right)$ as a division unit. In order to explore the effects of $h_{x}$ on the results of the experiment, we have also done a comparative experiment.

**Figure 15:**
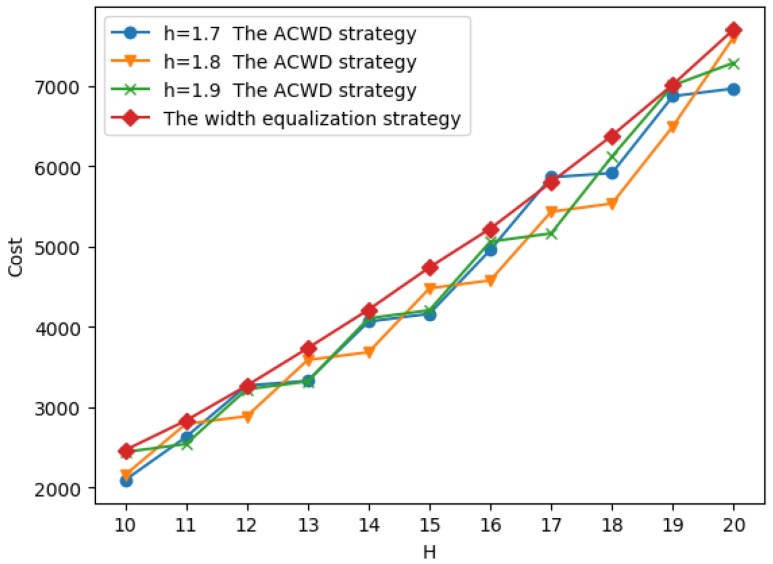
Comparison when 10≤H≤20.

We take different values of $h_{x}$ to experiment in the interval [1.7,1.9], and compare with the result of the equipartition placement strategy. As shown in Figure 14, We find that when the annulus barrier width H≤5, for different $h_{x}$, the experimental results are generally similar, and it is also similar to the equipartition placement strategy. However, as shown in Figure 15, with the increase of the annulus barrier width *H*, the influence of the $h_{x}$ on the effect of the experiment is good and bad, and there is no obvious rule. But it is better than the equipartition placement strategy.

## 6. Conclusions

In this paper, we studied the minimum cost BR sensor placement algorithm to construct a circular BR coverage with a predefined breadth and detection threshold. First, we investigate the Cassini oval sensing models and discuss a variety of barrier coverage cases. We discovered and proved the optimized BR placement patterns and sequence on a circular ring. Second, we further studied the optimal BR placement on a circular barrier with a predefined breadth. We proposed two division strategies, circular equipartition strategy and an adaptive segmentation strategy, to segment an annulus ring to several adjacent sub-rings with appropriate width so as to ensure the minimum cost annulus BR barrier coverage with required detection threshold. Finally, we proposed optimal placement algorithms for minimum cost placement of BR sensor for annulus barrier coverage with required width and detection threshold. We validated the effectiveness of the proposed algorithms through extensive simulation experiments.

In our paper, we proposed an approximate optimal solution that mainly adopts the circular transverse sensor deployment method. In the future works, we will further study more complex longitudinal sensor placement method to optimize the bistatic radar sensors deployment. Second, we will consider to investigating the deployment problems in the heterogeneous bistatic radar sensor network. Third, the fault-tolerant problem in perimeter barrier deployment is also worth to study for high reliable coverage.

## Figures and Tables

**Table 1:** Symbols and descriptions.

Symbols	Descriptions
$T_{i}\left(R_{j}\right)$	The *i*th BR transmitter (*j*th receiver)
*H*	The width of circular barrier coverage
ε	The detection threshold of SNR
l(A)	The detectability of *A*
$l_{\max}$	The maximum threshold
d(T,R)	The distance between *T* and *R*
$C_{T}\left(C_{R}\right)$	The unit price of BR transmitter(receiver)
M(N)	The number of BR transmitter(receiver)
*q*	The number of circles
*S*	The deployment sequence of BR sensors
$P_{i}^{n}$	The optimal deployment sequence of BRs
*h*	The width of circle
$R_{\min}$	The inner radius of circle
$R_{\max}$	The outer radius of circle
*r*	$\frac{R_{\min}+R_{\max}}{2}$
$\theta_{i}$	The center angle of a circle
$\theta_{\max}$	The maximum angle which the sequence $P_{i}^{n}$ can cover
$d_{n}$	The effective coverage width of $P_{i}^{n}$
$L_{n}$	The effective coverage length of $P_{i}^{n}$
μ	The upper bound of the number of circles

**Table 2:** Parameter setting.

Parameter	Value
$l_{\max}^{2}$	3
*h*	(0, 2.4)
*q*	[$\frac{H}{\sqrt{2}l_{\max}}$, $\frac{H}{0.5}$]
$R_{m}$	3
*H*	[1, 20]
$C_{T}$	50
$C_{R}$	1, 5, 25
λ	[1, 50]

**Figure A1:**
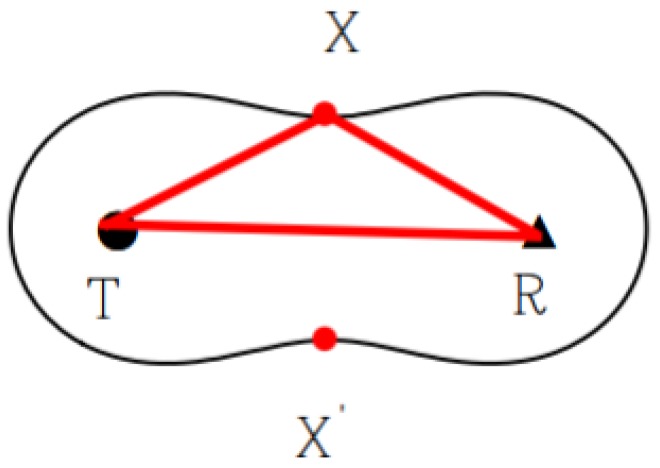
The schematic diagram of the Theorem 1.

**Figure A2:**
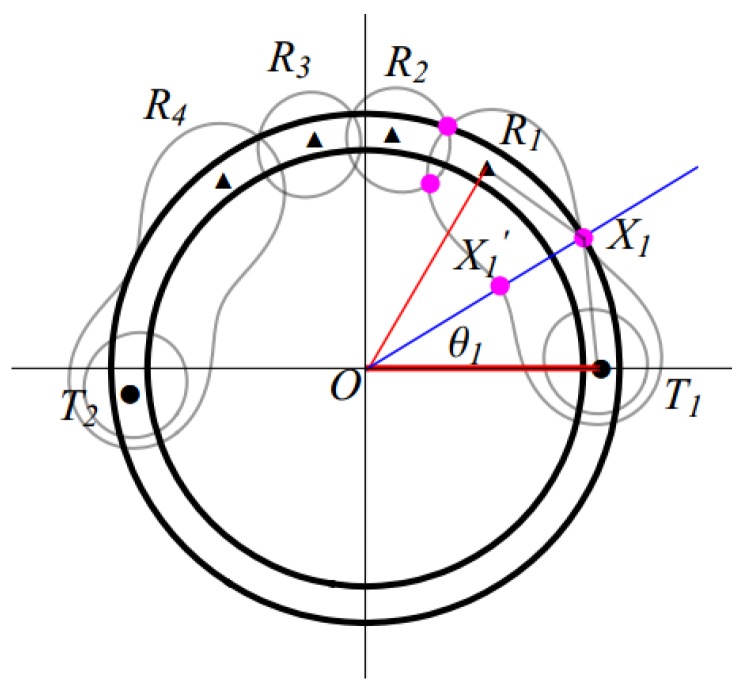
The schematic diagram of $\theta_{1}$ in Theorem 2.

**Figure A3:**
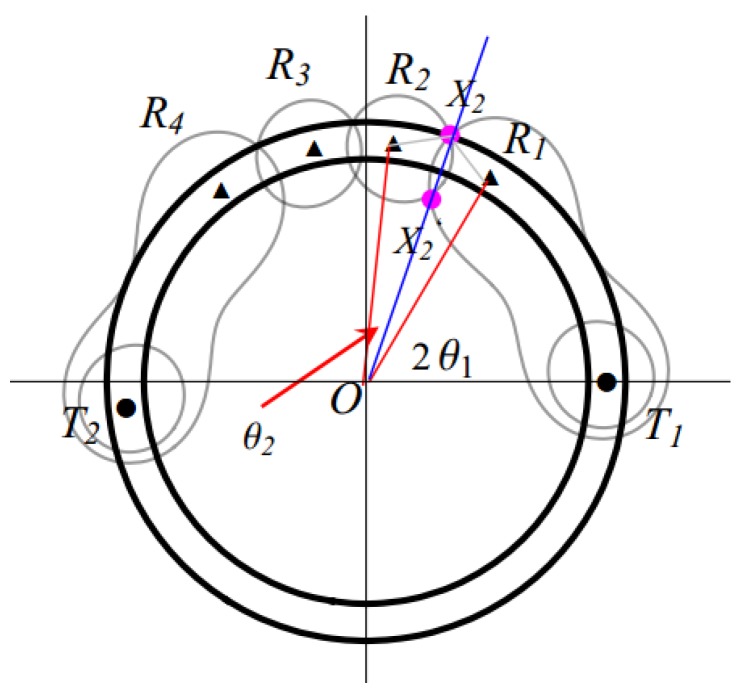
The schematic diagram of $\theta_{2}$ in Theorem 2.

**Figure A4:**
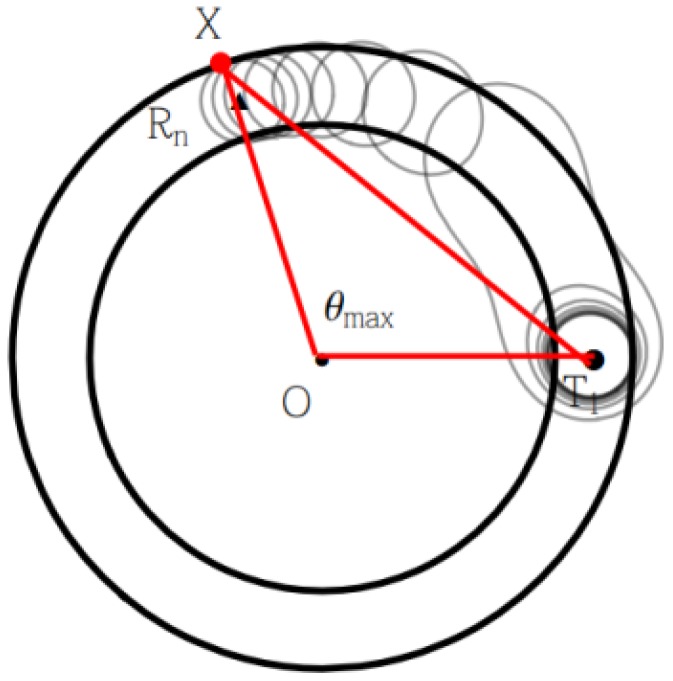
The schematic diagram of $n_{\max}$ in Theorem 3.

**Figure A5:**
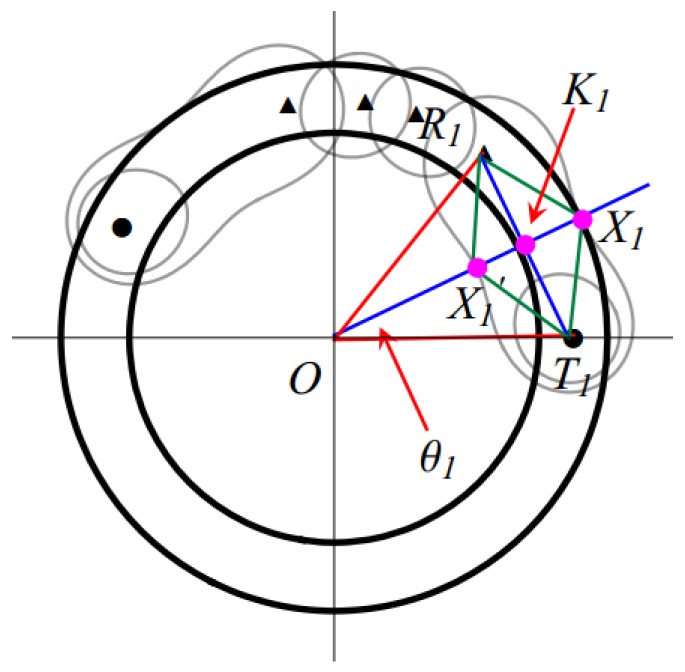
The width of $T_{1}-R_{1}$ in Theorem 4.

**Figure A6:**
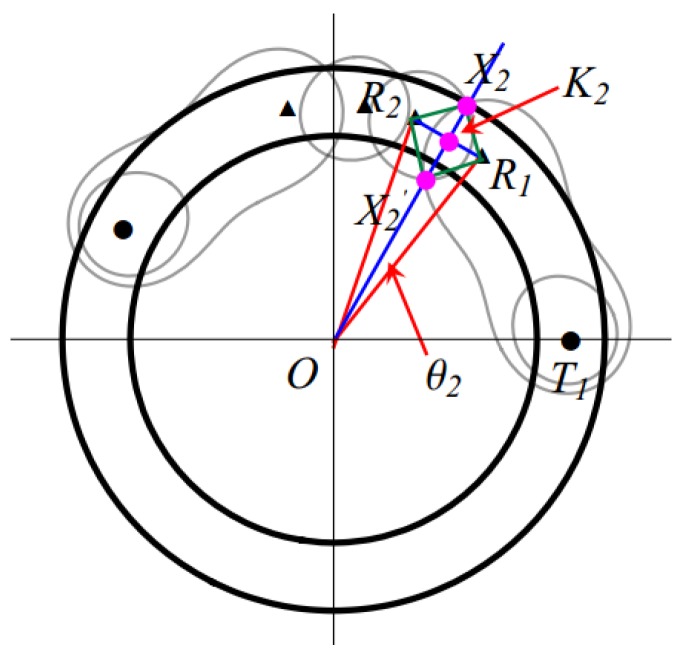
The width of $T_{1}-R_{2}$ in Theorem 4.

**Figure A7:**
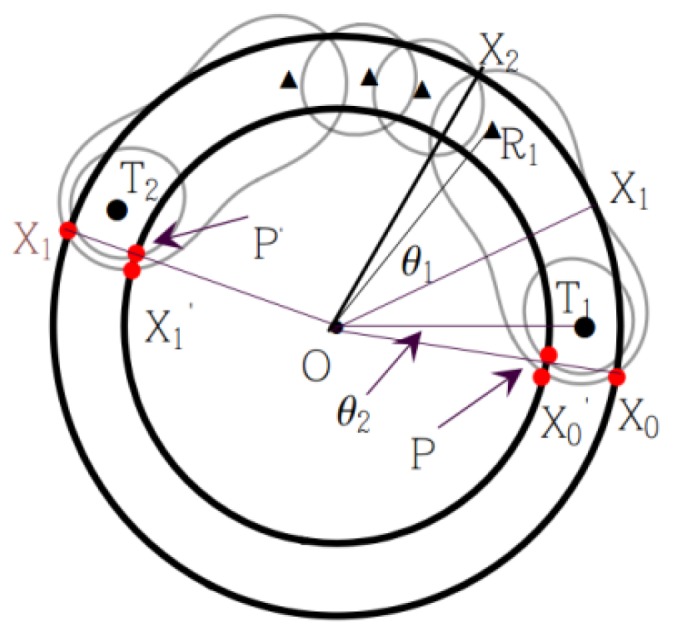
The schematic diagram of the effective coverage length in Theorem 6.

## Appendix A

**Proof** **of** **Theorem** **1.** As shown in Figure A1, the coverage area of the BR sensor is surrounded by the Cassini oval line, and it is symmetrical. The perpendicular bisector of the line segment $\bar{TR}$ cross the regional boundary in point *X* and point $X^{\prime }$. We know that if a point *A* can be detected by sensors, it is necessary to satisfy the condition $∥TA∥\cdot ∥RA∥\leq l_{\max}^{2}$. The point *X* is on the border of the covering area, so there has $∥TX∥\cdot ∥RX∥=l_{\max}^{2}$, equivalenting to $\frac{K}{{∥TX∥}^{2}\cdot {∥RX∥}^{2}}=\varepsilon$. In the same way, the point $X^{\prime }$ also satisfies this condition. □

**Proof** **of** **Theorem** **2.** For the sequence $P_{i}^{n}$, we are used $\left(2\theta_{1},2\theta_{2},\ldots ,2\theta_{n+1}\right)$ to represent the combination of its center angle, where $\theta_{1}=\frac{1}{2}∠T_{i}OR_{1}$, $\theta_{2}=\frac{1}{2}∠R_{1}OR_{2}$, …, $\theta_{n}=\frac{1}{2}∠R_{n-1}OR_{n}$, $\theta_{n+1}=\frac{1}{2}∠R_{n}OT_{i+1}$. Here we define the angle sum of a single covering sequence as $Sum=\sum_{i=1}^{n+1}2\theta_{i}$. In the worst case, we only need to use a single covering sequence to cover the circle, so Sum=2π, but normally, Sum<2π. Therefore, $\sum_{i=1}^{n+1}2\theta_{i}\leq 2\pi$. Due to the particularity of circle and the Cassini oval, the sensors sequence $P_{i}^{n}$ is symmetrical, on the axis symmetry of the center line of the circular arc ^TiTi+1. As a result, there has the following properties: (1) if *n* is odd, then $\theta_{1}=\theta_{n+1}$, $\theta_{2}=\theta_{n}$, …, $\theta_{\frac{n+1}{2}}=\theta_{\frac{n+1}{2}+1}$; (2) if *n* is even, then $\theta {}_{1}=\theta_{n+1}$, $\theta_{2}=\theta_{n}$, …, $\theta_{\frac{n}{2}}=\theta_{\frac{n}{2}+2}$, $\theta_{\frac{n}{2}+1}=\theta_{\frac{n}{2}+1}$. Next, we give the specific calculation of the angle $\theta_{i}$. For $\theta_{1}$, as shown in Figure A2, we have proved that the covering model of waist shape can best meet our coverage requirements. Next, we will determine the angle $\theta_{1}$. We have got the detectability of the point $X_{1}$, it is $l_{\max}^{2}$. We know $∥T_{1}X_{1}∥=∥R_{1}X_{1}∥$, $∠T_{1}OX_{1}=∠R_{1}OX_{1}=\theta_{1}$, $\Delta T_{1}OX_{1}≅\Delta R_{1}OX_{1}\left(SSS\right)$, the signal-to-noise ratio of the point $X_{1}$ is ε, then we can list the equation $\frac{K}{{∥T_{1}X_{1}∥}^{2}\cdot {∥R_{1}X_{1}∥}^{2}}=\varepsilon$. Introducing the concept of the detectability, it can be reduced to:

(A1) $$∥T_{1}X_{1}∥\cdot ∥R_{1}X_{1}∥={∥T_{1}X_{1}∥}^{2}=l_{\max}^{2}$$

then using the cosine equation we can obtain the following equation:

(A2) $${∥T_{1}X_{1}∥}^{2}={∥T_{1}O∥}^{2}+{∥X_{1}O∥}^{2}-2∥T_{1}O∥\cdot ∥X_{1}O∥\cos\theta_{1}$$

Jointing Equations (6) and (7), we can get: $\theta_{1}=\arccos\frac{r^{2}+R_{\max}^{2}-l_{\max}^{2}}{2rR_{\max}}$. We guarantee that $0\;\leq \;\frac{r^{2}+R_{\max}^{2}-l_{\max}^{2}}{2rR_{\max}}<1$, otherwise, the coverage will be meaningless. For $\theta_{2}$, as shown in Figure A3, we found that the cover model which constituted by the second receiver $R_{2}$ and the transmitter $T_{1}$ can be the waist shaped or two separate parts, as shown in Figure 1a,c in the paper. In order not to lose the generality, we assume that the model $T_{1}-R_{2}$ is two separate parts. Then let’s give the location of the receiver $R_{2}$. As shown in Figure A3, the coverage area of the sensor $T_{1}-R_{1}$ intersects with the circle at point $X_{2}$. In order to meet the coverage, the coverage area of the sensor $T_{1}-R_{2}$ should intersect with the former at the point $X_{2}$. At this point, we can get the signal-to-noise ratio of $X_{2}$ is ε, the detectability is $l_{\max}^{2}$, so we have the equation $∥T_{1}X_{2}∥\cdot ∥R_{1}X_{2}∥=l_{\max}^{2}$, $∥T_{1}X_{2}∥\cdot ∥R_{2}X_{2}∥=l_{\max}^{2}$, and we can get $∥R_{1}X_{2}∥=∥R_{2}X_{2}∥$. At the same time, we get $\Delta R_{1}OX_{2}≅\Delta R_{2}OX_{2}(SSS)$, $∠R_{1}OX_{2}=∠R_{2}OX_{2}=\theta_{2}$. We know that the detectability at the point $X_{2}$ is $l_{\max}^{2}$, then get $∥T_{1}X_{2}∥\cdot ∥R_{1}X_{2}∥=l_{\max}^{2}$, using the cosine theorem can get ${∥T_{1}X_{2}∥}^{2}=r^{2}+R_{\max}^{2}-2rR_{\max}\cdot \cos\left(2\theta_{1}+\theta_{2}\right)$, ${∥R_{1}X_{2}∥}^{2}=r^{2}+R_{\max}^{2}-2rR_{\max}\cdot \cos\theta_{2}$. Then taking the two equation into the upper, it can be reduced to:

(A3) $$\begin{array}{c} 4r^{2}R_{\max}^{2}\cos^{2}\left(\theta_{1}+\theta_{2}\right)-4rR\cdot \left(r^{2}+R_{\max}^{2}\right)\cdot \cos\theta_{1}\cdot \cos\left(\theta_{1}+\theta_{2}\right)+\\ 4r^{2}R_{\max}^{2}\left(\cos^{2}\theta_{1}-1\right)+{\left(r^{2}+R_{\max}^{2}\right)}^{2}-l_{\max}^{4}=0 \end{array}$$

For solving this equation, at first, we assume that the left of the equation is two polynomial functions *f*, the independent variable is $\cos(\theta_{1}+\theta_{2})$. In the previous paper, we have got $\sum_{i=1}^{n+1}2\theta_{i}\leq 2\pi$, then $\sum_{i=1}^{n+1}\theta_{i}\leq \pi$. Because $\cos(\theta_{1}+\theta_{2})$ monotonous decreasing in the interval [0,π], the independent variable decreases monotonously in the interval [0,π]. We can get the symmetric axis of function *f* is $x_{m}=\frac{\left(r^{2}+R_{\max}^{2}\right)\cos\theta_{1}}{2rR_{\max}}$, and we can get $x_{m}>\cos\theta_{1}$, so there has $x_{m}>\cos\theta_{1}>\cos\left(\theta_{1}+\theta_{2}\right)$, it is that in the solution of the equation f=0, the solution we need is on the left of the symmetric axis. Then combined with the Equation (8), we can get:

(A4) $$\Delta ={\left(R_{\max}+r\right)}^{2}{\left(R_{\max}-r\right)}^{2}\left(\cos^{2}\theta_{1}-1\right)+l^{4}$$

(A5) $$x_{1}\;=\;\frac{\left(R_{\max}^{2}\;+\;r^{2}\right)\cos\theta_{1}\;+\;\sqrt{{\left(R_{\max}\;+\;r\right)}^{2}{\left(R_{\max}\;-\;r\right)}^{2}\left(\cos^{2}\theta_{1}\;-\;1\right)\;+\;l^{4}}}{2rR_{\max}}$$

(A6) $$x_{2}\;=\;\frac{\left(R_{\max}^{2}\;+\;r^{2}\right)\cos\theta_{1}\;-\;\sqrt{{\left(R_{\max}\;+\;r\right)}^{2}{\left(R_{\max}\;-\;r\right)}^{2}\left(\cos^{2}\theta_{1}\;-\;1\right)\;+\;l^{4}}}{2rR_{\max}}$$

We take $x_{2}=\cos\left(\theta_{1}+\theta_{2}\right)$. We ensure Δ>0, it is $l_{\max}^{4}>{\left(R_{\max}+r\right)}^{2}\cdot {\left(R_{\max}-r\right)}^{2}\cdot \left(1-\cos^{2}\theta_{1}\right)$, at the same time, we ensure $-1<x_{2}<1$. In summary, we get:

(A7) $$\theta_{2}=\arccos\left(\frac{(R_{\max}^{2}+r^{2})\cos\theta_{1}-\sqrt{{\left(R_{\max}+r\right)}^{2}{\left(R_{\max}-r\right)}^{2}(\cos^{2}\theta_{1}-1)+l_{\max}^{4}}}{2rR_{\max}}\right)-\theta_{1}$$

For $\theta_{3}$, at the same as $\theta_{2}$. We can get $∥R_{2}X_{3}∥=∥R_{3}X_{3}∥$, $\Delta R_{2}OX_{3}\;=\;\Delta R_{3}OX_{3}(SSS)$, $∠R_{2}OX_{3}\;=\;∠R_{3}OX_{3}=\theta_{3}$, the detectability of $X_{3}$ is $l_{\max}^{2}$, $∥T_{1}X_{3}∥\cdot ∥R_{2}X_{3}∥=l_{\max}^{2}$, joints all, we can get:

(A8) $$\Delta ={\left(R_{\max}+r\right)}^{2}{\left(R_{\max}-r\right)}^{2}\left[\cos^{2}\left(\theta_{1}+\theta_{2}\right)-1\right]+l_{\max}^{4}$$

(A9) $$x_{3}=\frac{\left(R_{\max}^{2}+r^{2}\right)\cos\left(\theta_{1}+\theta_{2}\right)-\sqrt{{\left(R_{\max}+r\right)}^{2}{\left(R_{\max}-r\right)}^{2}\left[\cos^{2}\left(\theta_{1}+\theta_{2}\right)-1\right]+l_{\max}^{4}}}{2rR_{\max}}$$

We ensure Δ>0, $-1<x_{3}<1$, so we get:

(A10) $$\begin{array}{c} \theta_{3}=\arccos\left(\frac{\left(R_{\max}^{2}+r^{2}\right)\cos\left(\theta_{1}+\theta_{2}\right)-\sqrt{{\left(R_{\max}+r\right)}^{2}{\left(R_{\max}-r\right)}^{2}\left[\cos^{2}\left(\theta_{1}+\theta_{2}\right)-1\right]+l_{\max}^{4}}}{2rR}\right)\\ \;\;-(\theta_{1}+\theta_{2}) \end{array}$$

In the same way, analogous, we can get from $\theta_{4}$ to $\theta_{n}$. By observing, we can draw the rule, so we get $\theta_{i}$, i>1, it is:

(A11) $$\begin{array}{c} \theta_{i}=\arccos\left(\frac{(R_{\max}^{2}+r^{2})\cos\sum_{i=1}^{x-1}\theta_{i}-\sqrt{{\left(R_{\max}+r\right)}^{2}{\left(R_{\max}-r\right)}^{2}(\cos^{2}\sum_{i=1}^{x-1}\theta_{i}-1)+l_{\max}^{4}}}{2rR_{\max}}\right)\\ \;\;-\sum_{i=1}^{x-1}\theta_{i},i>1 \end{array}$$

To sum up, we give the calculation equation of the center angle θ. □

**Proof** **of** **Theorem** **3.** Figure A4 gives a partial schematic diagram of the sequence $P_{i}^{n}$. We assume point *X* as the intersection of the sensors’ coverage area and the circle boundary. We found that with the increase of the number of receivers, the coverage area of single sensor decreases continuously, and the point *X* is more and more close to the outside boundary of the circle. When *n* is taken to the maximum value $n_{\max}$, the coverage area of the sensors is tangent to the outer boundary of the circle at the point *X*, and the connection between the point *O* and the point *X* is over the point $R_{n}$ at this time. Assume that $∠T_{1}OX=\theta_{\max}$, it is obvious that the maximum center angle of the sequence $P_{i}^{n}$ is $2\theta_{\max}$. At the same time, the detectability of the point *X* is $l_{\max}^{2}$, so there has $∥T_{1}X∥\cdot ∥R_{n}X∥=l_{\max}^{2}$. Using the cosine theorem, we can get ${∥T_{1}X∥}^{2}={∥T_{1}O∥}^{2}+{∥XO∥}^{2}-2∥T_{1}O∥\cdot ∥XO∥\cdot \cos\theta_{\max}$. Jointing all, we can get $\theta_{\max}=\arccos\left(\frac{\left(R_{\max}^{2}+r^{2}\right)\cdot {\left(R_{\max}-r\right)}^{2}-l_{\max}^{4}}{2rR_{\max}\cdot {\left(R_{\max}-r\right)}^{2}}\right)$. Theorem 2 gives the concept of the center angle of the circle, so we get $\sum_{i=1}^{n_{\max}+1}2\theta_{i}=2\theta_{\max}$. We can calculate the angle $\theta_{i}$ in turn, until the sum is equal to $2\theta_{\max}$, then the *n* at this moment is the largest number of receivers $n_{\max}$. □

**Proof** **of** **Theorem** **4.** In the paper, we use $2∥X_{3}K_{3}∥$ as the approximation length of the line segment $\bar{X_{3}{X_{3}}^{\prime }}$ and as the effective coverage width. Here we extend the value *n* to $n_{\max}$, and give the calculation equation of effective coverage width. For the sensor pair $T_{1}-R_{1}$, as shown in Figure A5, we connect the point $T_{1}$ and the point $R_{1}$. We have obtained $∠T_{1}OX_{1}=∠R_{1}OX_{1}=\theta_{1}$, so $\Delta T_{1}OK_{1}≅\Delta R_{1}OK_{1}(SAS)$. Therefore, there has $∠R_{1}K_{1}O=∠T_{1}K_{1}O=90^{\circ }$, $∥R_{1}K_{1}∥=∥T_{1}K_{1}∥$, so we can know that the line $X_{1}O$ is the perpendicular bisector of the line segment $\bar{T_{1}R_{1}}$, and can get $∥X_{1}K_{1}∥=R_{\max}-r\cdot \cos\theta_{1}$ further. At the same time, we have $∥T_{1}X_{1}∥\cdot ∥R_{1}X_{1}∥=l_{\max}^{2}$, $∥T_{1}{X_{1}}^{\prime }∥\cdot ∥R_{1}{X_{1}}^{\prime }∥=l_{\max}^{2}$, $∥T_{1}X_{1}∥=∥R_{1}X_{1}∥$, $∥T_{1}{X_{1}}^{\prime }∥=∥R_{1}X{{}_{1}}^{\prime }∥$, so we can get $∥T_{1}X_{1}∥=∥T_{1}{X_{1}}^{\prime }∥$, and the quadrilateral $T_{1}X_{1}R_{1}{X_{1}}^{\prime }$ is a diamond, so $∥X_{1}{X_{1}}^{\prime }∥\;=\;2∥X_{1}K_{1}∥=d_{1}=2\left(R_{\max}-r\cdot \cos\theta_{1}\right)$. Here, the two times value just equal to the actual coverage width, because there has $∥X_{n}{X_{n}}^{\prime }∥\geq 2∥X_{n}K_{n}∥$. For the sensor pair $T_{1}-R_{2}$, as shown in Figure A6, similarly, we can get that the line $X_{2}O$ is the perpendicular bisector of the line segment $\bar{R_{1}R_{2}}$, then we prove the point ${X_{2}}^{\prime }$ is on the line $X_{2}O$, that is $X_{2}$, ${X_{2}}^{\prime }$, *O*, three points are collinear. We know that the detectability of the point $X{}_{2}^{\prime }$ is $l_{\max}^{2}$, so $∥T_{1}{X_{2}}^{\prime }∥\cdot ∥R_{1}{X_{2}}^{\prime }∥=l_{\max}^{2}$, $∥T_{1}{X_{2}}^{\prime }∥\cdot ∥R_{2}{X_{2}}^{\prime }∥=l_{\max}^{2}$. We get $∥R_{1}{X_{2}}^{\prime }∥=∥R_{2}{X_{2}}^{\prime }∥$, it is that the point ${X_{2}}^{\prime }$ is on the perpendicular bisector of the line segment $\bar{R_{1}R_{2}}$. Because the line $X_{2}O$ is the perpendicular bisector of the line segment $\bar{R_{1}R_{2}}$, the point ${X_{2}}^{\prime }$ is on the line $X_{2}O$, the point $X_{2}$, ${X_{2}}^{\prime }$, *O* are collinear. At the same time, we can get $∥T_{1}X_{2}∥>∥T_{1}{X_{2}}^{\prime }∥$, also because of $∥T_{1}X_{2}∥\cdot ∥R_{1}X_{2}∥=l_{\max}^{2}$, $∥T_{1}{X_{2}}^{\prime }∥\cdot ∥R_{1}{X_{2}}^{\prime }∥=l_{\max}^{2}$, we can get $∥R_{1}X_{2}∥<∥R_{1}{X_{2}}^{\prime }∥$, $∥K_{2}{X_{2}}^{\prime }∥\;>\;∥K_{2}X_{2}∥$, $∥X_{2}{X_{2}}^{\prime }∥>2∥X_{2}K_{2}∥=d_{2}=2\left(R_{\max}-r\cdot \cos\theta_{2}\right)$. In the same way, Calculating the $d_{i}$ in turn, we can get the induction $d_{n}=2\left(R-r\cdot \cos\theta_{\frac{n}{2}+1}\right)$, $1\leq n\leq n_{\max}$. □

**Proof** **of** **Theorem** **5.** In Theorem 4, we have given the effective coverage width of the sequence $P_{i}^{n}$ and get the actual coverage width $∥X_{n}{X_{n}}^{\prime }∥\geq 2∥X_{n}K_{n}∥=d_{n}$. To prove the reliability of the coverage, we only need to prove $d_{n}>h$, where *h* is the width of the circle. We found that the expression of the two times value is $d_{n}=2\left(R_{\max}-r\cdot \cos\theta_{\frac{n}{2}+1}\right)$, the width of the circle is $h=2(R_{\max}-r)$, then there has $d_{n}-h=2r\cdot \left(1-\cos\theta_{\frac{n}{2}+1}\right)>0$, so we can get the two times value meets the requirements.

**Proof** **of** **Theorem** **6.** As shown in Figure A7, we connect the point *O* and the point $X_{0}$, intersects the inner boundary of the circle at the point *P*. It is visible that the line segment $\bar{X_{0}P}$ is in the circle, and we use this line segment as the starting position of the sum of the center angle. For the sequence $P_{i}^{1}$, first of all, due to the symmetry of the covering area, we can get $∠X_{0}OT_{1}\;=\;∠X_{2}OR_{1}=\theta_{2}$, and the center angle $∠X_{0}OX_{2}=2\left(2\theta_{1}+\theta_{2}\right)$, it is that the coverage length is $L_{1}\;=\;2r\cdot \left(2\theta_{1}+\theta_{2}\right)$. For the sequence $P_{i}^{2}$, we can also get the coverage length $L_{2}=2r\cdot \left(2\theta_{1}+\theta_{2}+\theta_{2}\right)$. For the sequence $P_{i}^{3}$, the coverage length is $L_{3}=2r\cdot \left(2\theta_{1}+2\theta_{2}+\theta_{2}\right)$. For the sequence $P_{i}^{4}$, the coverage length is $L_{4}=2r\cdot \left(2\theta_{1}+2\theta_{2}+\theta_{3}+\theta_{2}\right)$. Calculate in turn. For the sequence $P_{i}^{n}$, the coverage length can becalculated as Equation 9. □

**Proof** **of** **Theorem** **7.** For the sequence $S=(T_{1},R^{n_{1}},T_{2},R^{n_{2}},T_{3})$ in the paper, $n_{1}$ is odd, $n_{2}$ is even, and we can calculate the center angle of its coverage is

(A12) $$\begin{array}{cc} \psi_{1} & =2\left(2\sum_{i=1}^{\frac{n_{1}+1}{2}}\theta_{i}+2\sum_{i=1}^{\frac{n_{2}}{2}}\theta_{i}+\theta_{\frac{n_{2}}{2}+1}+\theta_{2}\right)\\ & =\left\{\begin{array}{c} 2\left(4\sum_{i=1}^{\frac{n_{2}}{2}}\theta_{i}+\theta_{\frac{n_{2}}{2}+1}+\theta_{2}\right),\;n_{2}-n_{1}=1\\ 2\left(4\sum_{i=1}^{\frac{n_{1}+1}{2}}\theta_{i}+2\sum_{i=\frac{n_{1}+1}{2}+1}^{\frac{n_{2}}{2}}\theta_{i}+\theta_{\frac{n_{2}}{2}+1}+\theta_{2}\right),\;n_{2}-n_{1}>1 \end{array}\right. \end{array}.$$

For the sequence $S^{\prime }=\left(T_{1},R^{\frac{n_{1}+n_{2}-1}{2}},T_{2},R^{\frac{n_{1}+n_{2}+1}{2}},T_{3}\right)$, we categorize:

(1) When $n_{2}-n_{1}=1$, $\frac{n_{1}+n_{2}-1}{2}$ is odd, $\frac{n_{1}+n_{2}+1}{2}$ is even, we can get:
  (A13) $$\begin{array}{cc} \psi_{2} & =2\left(2\sum_{i=1}^{\frac{n_{1}+n_{2}+1}{4}}\theta_{i}+2\sum_{i=1}^{\frac{n_{1}+n_{2}+1}{4}}\theta_{i}+\theta_{\frac{n_{1}+n_{2}+1}{4}+1}+\theta_{2}\right)\\ & =2\left(4\sum_{i=1}^{\frac{n_{1}+n_{2}+1}{4}}\theta_{i}+\theta_{\frac{n_{1}+n_{2}+1}{4}+1}+\theta_{2}\right)\\ & =2\left(4\sum_{i=1}^{\frac{n_{2}}{2}}\theta_{i}+\theta_{\frac{n_{2}}{2}+1}+\theta_{2}\right) \end{array}$$
  At this point, $\psi_{1}=\psi_{2}$.
(2) When $n_{2}-n_{1}>1$, $\frac{n_{1}+n_{2}-1}{2}$ may be odd or even, so we continue to categorize.
  *a*. If $\frac{n_{1}+n_{2}-1}{2}$ is odd, $\frac{n_{1}+n_{2}+1}{2}$ is even, then we can get
  (A14) $$\psi_{2}=2\left(4\sum_{i=1}^{\frac{n_{1}+n_{2}+1}{4}}\theta_{i}+\theta_{\frac{n_{1}+n_{2}+5}{4}}+\theta_{2}\right).$$
  And then we compare $\psi_{1}$ and $\psi_{2}$:
  (A15) $$\begin{array}{cc} \psi_{2}-\psi_{1} & =2\left(4\sum_{i=1}^{\frac{n_{1}+n_{2}+1}{4}}\theta_{i}+\theta_{\frac{n_{1}+n_{2}+5}{4}}+\theta_{2}\right)-\\ & \;2\left(4\sum_{i=1}^{\frac{n_{1}+1}{2}}\theta_{i}+2\sum_{i=\frac{n_{1}+1}{2}+1}^{\frac{n_{2}}{2}}\theta_{i}+\theta_{\frac{n_{2}}{2}+1}+\theta_{2}\right)\\ & =2\left(4\sum_{i=\frac{n_{1}+1}{2}+1}^{\frac{n_{1}+n_{2}+1}{4}}\theta_{i}-2\sum_{i=\frac{n_{1}+1}{2}+1}^{\frac{n_{2}}{2}}\theta_{i}+\theta_{\frac{n_{1}+n_{2}+5}{4}}-\theta_{\frac{n_{2}}{2}+1}\right)\\ & =2\left(\underset{A}{\underset{︸}{2\sum_{i=\frac{n_{1}+1}{2}+1}^{\frac{n_{1}+n_{2}+1}{4}}\theta_{i}}}-\underset{B}{\underset{︸}{2\sum_{i=\frac{n_{1}+n_{2}+5}{4}}^{\frac{n_{2}}{2}}\theta_{i}}}+\underset{C}{\underset{︸}{\theta_{\frac{n_{1}+n_{2}+5}{4}}}}-\underset{D}{\underset{︸}{\theta_{\frac{n_{2}}{2}+1}}}\right) \end{array}$$
  Because $\frac{n_{1}+1}{2}<\frac{n_{1}+n_{2}+1}{4}$, so we get the second step, at the same time, $\frac{n_{1}+n_{2}+1}{4}<\frac{n_{2}}{2}$, so, we get the last step. We see that in the last step, it contains *A*, *B*, *C*, *D*, four parts, only need to compare the size of the four parts can judge the size of $\psi_{1}$ and $\psi_{2}$. We get $\frac{n_{1}+n_{2}+1}{4}-\left(\frac{n_{1}+1}{2}+1\right)=\frac{n_{2}-n_{1}-5}{4}$, $\frac{n_{2}}{2}-\frac{n_{1}+n_{2}+5}{4}=\frac{n_{2}-n_{1}-5}{4}$. Therefore, the *A* and *B* contain the same number of angles. At the same time, Theorem 2 gets the value of the angle $\theta_{i}$, and we can find that $\theta_{i}$ decreases with *i* increase. Compared to the first angle of *A* and *B*, we get $\frac{n_{1}+1}{2}+1-\frac{n_{1}+n_{2}+5}{4}=\frac{n_{1}-n_{2}}{4}<0$, that is $\theta_{\frac{n_{1}+1}{2}+1}>\theta_{\frac{n_{1}+n_{2}+5}{4}}$. Compared to the last angle of *A* and *B*, we get $\frac{n_{1}+n_{2}+1}{4}-\frac{n_{2}}{2}=\frac{n_{1}-n_{2}+1}{4}<0$, so A>B. We can also get $\frac{n_{1}+n_{2}+5}{4}-\left(\frac{n_{2}}{2}+1\right)=\frac{n_{1}-n_{2}+1}{4}<0$, so C>D. In summary, $\psi_{2}>\psi_{1}$.
  *b*. When $\frac{n_{1}+n_{2}-1}{2}$ is even, $\frac{n_{1}+n_{2}+1}{2}$ is odd, then we get
  (A16) $$\begin{array}{c} \psi_{2}=2\left(2\sum_{i=1}^{\frac{n_{1}+n_{2}+3}{4}}\theta_{i}+2\sum_{i=1}^{\frac{n_{1}+n_{2}-1}{4}}\theta_{i}+\theta_{\frac{n_{1}+n_{2}-1}{4}+1}+\theta_{2}\right)\\ \;=2\left(4\sum_{i=1}^{\frac{n_{1}+n_{2}+3}{4}}\theta_{i}-\theta_{\frac{n_{1}+n_{2}+3}{4}}+\theta_{2}\right) \end{array}$$
  We also compare $\psi_{1}$ and $\psi_{2}$:
  (A17) $$\begin{array}{cc} \psi_{2}-\psi_{1} & =2\left(4\sum_{i=1}^{\frac{n_{1}+n_{2}+3}{4}}\theta_{i}-\theta_{\frac{n_{1}+n_{2}+3}{4}}+\theta_{2}\right)-\\ & \;2\left(4\sum_{i=1}^{\frac{n_{1}+1}{2}}\theta_{i}+2\sum_{i=\frac{n_{1}+1}{2}+1}^{\frac{n_{2}}{2}}\theta_{i}+\theta_{\frac{n_{2}}{2}+1}+\theta_{2}\right)\\ & =2\left(4\sum_{i=\frac{n_{1}+1}{2}+1}^{\frac{n_{1}+n_{2}+3}{4}}\theta_{i}-\theta_{\frac{n_{1}+n_{2}+3}{4}}-2\sum_{i=\frac{n_{1}+1}{2}+1}^{\frac{n_{2}}{2}}\theta_{i}-\theta_{\frac{n_{2}}{2}+1}\right)\\ & =2\left(4\sum_{i=\frac{n_{1}+1}{2}+1}^{\frac{n_{1}+n_{2}-1}{4}}\theta_{i}+3\theta_{\frac{n_{1}+n_{2}+3}{4}}-2\sum_{i=\frac{n_{1}+1}{2}+1}^{\frac{n_{2}}{2}}\theta_{i}-\theta_{\frac{n_{2}}{2}+1}\right)\\ & =2\left(2\sum_{i=\frac{n_{1}+1}{2}+1}^{\frac{n_{1}+n_{2}-1}{4}}\theta_{i}+3\theta_{\frac{n_{1}+n_{2}+3}{4}}-2\sum_{i=\frac{n_{1}+n_{2}}{4}+3}^{\frac{n_{2}}{2}}\theta_{i}-\theta_{\frac{n_{2}}{2}+1}\right)\\ & =2\left(\underset{A}{\underset{︸}{2\sum_{i=\frac{n_{1}+1}{2}+1}^{\frac{n_{1}+n_{2}-1}{4}}\theta_{i}}}-\underset{B}{\underset{︸}{2\sum_{i=\frac{n_{1}+n_{2}+3}{4}+1}^{\frac{n_{2}}{2}}\theta_{i}}}+\underset{C}{\underset{︸}{\theta_{\frac{n_{1}+n_{2}+3}{4}}}}-\underset{D}{\underset{︸}{\theta_{\frac{n_{2}}{2}+1}}}\right) \end{array}$$
  Similarly, because of $\frac{n_{1}+n_{2}+3}{4}-\frac{n_{1}+1}{2}=\frac{n_{1}-n_{1}+1}{4}>0$, we get the second step, at the same time, because of $\frac{n_{1}+n_{2}-1}{4}-\frac{n_{2}}{2}=\frac{n_{1}-n_{2}-1}{4}<0$, we get the fourth step. Also because of $\frac{n_{1}+n_{2}-1}{4}-\left(\frac{n_{1}+1}{2}+1\right)=\frac{n_{2}}{2}-\left(\frac{n_{1}+n_{2}+3}{4}+1\right)$, so *A* and *B* contain the same number of angles. Because of $\frac{n_{1}+1}{2}+1<\frac{n_{1}+n_{2}+3}{4}+1$, $\frac{n_{1}+n_{2}-1}{4}<\frac{n_{2}}{2}$, so A>B. Also because of $\frac{n_{1}+n_{2}+3}{4}<\frac{n_{2}}{2}+1$, so C>D. In summary, $\psi_{2}>\psi_{1}$.

In summary, when $n_{2}-n_{1}>1$, $\psi_{2}>\psi_{1}$. □

**Proof** **of** **Theorem** **8.** We consider the boundary problem, and we need to determine how many receivers are needed to assist the remainder of the circle. First, we need to calculate the size of the circle region which the sequence $T_{i}-R^{k}-T_{i+1}$ can cover. In theorem 6, we give the related calculation methods and formulae. Here we consider the overlap case of the coverage area, and give the results after simple calculation. When k=0, $a{}_{0}=0$; When k=1, $a_{1}=2\theta_{1}$; When k=2, $a_{2}=2\theta_{1}+\theta_{2}$; In turn, it can be concluded that the induction is as Equation (10). Assume that the radian of the reminder arc is Lt, we consider the case of overlapping and calculate the size of the circle region which the sequence $T_{i}-R^{k}-T_{i+1}$ can cover, it is $a_{k}$. Then by comparison, the number of the receivers which are used to the boundary problems can be determined, that is (1) if $-2\theta_{2}\leq Lt\leq 0$, it does not need the extra receiver; (2) if $0<Lt\leq 2a_{n}-2\theta_{2}$, then the *x* sequences can not meet the coverage requirements, the remaining number of receivers are determined by the following equation: if $2a_{k-1}<Lt+2\theta_{2}\leq 2a_{k}$, it needs *k* receivers. □

## References

[1] Liu B., Dousse O., Wang J., Saipulla A. Strong barrier coverage of wireless sensor networks. Proceedings of the ACM Interational Symposium on Mobile Ad Hoc NETWORKING and Computing, MOBIHOC 2008.

[2] Yildiz E., Akkaya K., Sisikoglu E., Sir M.Y. (2014). Optimal Camera Placement for Providing Angular Coverage in Wireless Video Sensor Networks. IEEE Trans. Comput..

[3] Ammari H.M., Das S.K. (2011). Centralized and Clustered k-Coverage Protocols for Wireless Sensor Networks. IEEE Trans. Comput..

[4] Wang B., Xu H., Liu W., Liang H. (2013). A Novel Node Placement for Long Belt Coverage in Wireless Networks. IEEE Trans. Comput..

[5] Saipulla A., Westphal C., Liu B., Wang J. (2013). Barrier coverage with line-based deployed mobile sensors. Ad Hoc Netw..

[6] Chen J., Wang B., Liu W. (2015). Constructing Perimeter Barrier Coverage With Bistatic Radar Sensors. J. Network Comput. Appl..

[7] Wang B., Chen J., Liu W., Yang L.T. (2016). Minimum Cost Placement of Bistatic Radar Sensors for Belt Barrier Coverage. IEEE Trans. Comput..

[8] Chang H.Y., Kao L., Chang K.P., Chen C. Fault-Tolerance and Minimum Cost Placement of Bistatic Radar Sensors for Belt Barrier Coverage. Proceedings of the International Conference on Network and Information Systems for Computers.

[9] Ammari H.M. (2016). A Unified Framework for K-Coverage and Data Collection in Heterogeneous Wireless Sensor Networks. J. Parallel Distrib. Comput..

[10] Rahman M.O., Razzaque M.A., Hong C.S. Probabilistic Sensor Deployment in Wireless Sensor Network: A New Approach. Proceedings of the International Conference on Advanced Communication Technology.

[11] Lu Z., Li W.W., Pan M. (2015). Maximum Lifetime Scheduling for Target Coverage and Data Collection in Wireless Sensor Networks. IEEE Trans. Veh. Technol..

[12] Kim H., Kim D., Li D., Kwon S.S., Tokuta A.O., Cobb J.A. (2016). Maximum lifetime dependable barrier-coverage in wireless sensor networks. Ad Hoc Netw..

[13] Kumar S., Lai T.H., Arora A. (2007). Barrier Coverage with Wireless Sensors.

[14] Chen A., Kumar S., Lai T.H. (2010). Local Barrier Coverage in Wireless Sensor Networks. IEEE Trans. Mob. Comput..

[15] Tao D., Tang S., Zhang H., Mao X., Ma H. (2012). Strong barrier coverage in directional sensor networks. Comput. Commun..

[16] Zorbas D., Razafindralambo T. (2013). Prolonging network lifetime under probabilistic target coverage in wireless mobile sensor networks. Comput. Commun..

[17] Cheng C.F., Tsai K.T. (2012). Distributed Barrier Coverage in Wireless Visual Sensor Networks With *β*-QoM. IEEE Sens. J..

[18] Liu L., Zhang X., Ma H. Exposure-Path Prevention in Directional Sensor Networks Using Sector Model Based Percolation. Proceedings of the IEEE International Conference on Communications.

[19] Gong X., Zhang J., Cochran D., Xing K. Barrier coverage in bistatic radar sensor networks: Cassini oval sensing and optimal placement. Proceedings of the Fourteenth ACM International Symposium on Mobile Ad Hoc Networking and Computing.

[20] Tang L., Gong X., Wu J., Zhang J. (2013). Target Detection in Bistatic Radar Networks: Node Placement and Repeated Security Game. IEEE Trans. Wirel. Commun..

[21] Kim H., Ben-Othman J. (2017). HeteRBar: Construction of Heterogeneous Reinforced Barrier in Wireless Sensor Networks. IEEE Commun. Lett..

[22] Han R., Yang W., Zhang L. (2018). Achieving Crossed Strong Barrier Coverage in Wireless Sensor Network. Sensors.

[23] Kim H., Ben-Othman J., Cho S., Mokdad L. On Virtual Emotion Barrier in Internet of Things. Proceedings of the 2018 IEEE International Conference on Communications (ICC).

[24] Wang G., Cao G., La Porta T. A Bidding Protocol for Deploying Mobile Sensors. Proceedings of the IEEE International Conference on Network Protocols.

[25] Silvestri S. MobiBar: Barrier Coverage with Mobile Sensors. Proceedings of the Global Telecommunications Conference.

[26] Kong L., Liu X., Li Z., Wu M.Y. Automatic Barrier Coverage Formation with Mobile Sensor Networks. Proceedings of the IEEE International Conference on Communications.

[27] Kim H., Oh H., Bellavista P., Ben-Othman J. (2017). Constructing Event-driven Partial Barriers with Resilience in Wireless Mobile Sensor Networks. J. Netw. Comput. Appl..

[28] Wang Z., Chen H., Cao Q., Qi H., Wang Z., Wang Q. (2017). Achieving location error tolerant barrier coverage for wireless sensor networks. Comput. Netw. Int. J. Comput. Telecommun. Netw..

[29] Willis N.J., Griffiths H.D. (2008). Advances in bistatic radar (Willis, N.J. and Griffiths, H.D., Eds.; 2007) [Book Review]. IEEE Aerosp. Electron. Syst. Mag..

[30] Liang J., Liang Q. Orthogonal Waveform Design and Performance Analysis in Radar Sensor Networks. Proceedings of the Military Communications Conference, MILCOM.

[31] Ly H.D., Liang Q. Collaborative Multi-Target Detection in Radar Sensor Networks. Proceedings of the IEEE Military Communications Conference, MILCOM 2007.

[32] Yang Q., He S., Chen J. Energy-efficient area coverage in bistatic radar sensor networks. Proceedings of the IEEE Global Communications Conference.

